# Synthesis, Antiproliferative Activity and Radical Scavenging Ability of 5-*O*-Acyl Derivatives of Quercetin

**DOI:** 10.3390/molecules26061608

**Published:** 2021-03-14

**Authors:** Stephen Lo, Euphemia Leung, Bruno Fedrizzi, David Barker

**Affiliations:** 1 School of Chemical Sciences, University of Auckland, 23 Symonds Street, Auckland 1010, New Zealand; slo023@aucklanduni.ac.nz (S.L.); b.fedrizzi@auckland.ac.nz (B.F.); 2 Auckland Cancer Society Research Centre, University of Auckland, Auckland 1023, New Zealand; e.leung@auckland.ac.nz; 3 MacDiarmid Institute for Advanced Materials and Nanotechnology, Wellington 6012, New Zealand

**Keywords:** quercetin, antiproliferative, colon cancer, breast cancer, radical scavenging

## Abstract

Quercetin is a flavonoid that is found in many plant materials, including commonly eaten fruits and vegetables. The compound is well known for its wide range of biological activities. In this study, 5-*O*-acyl derivatives of quercetin were synthesised and assessed for their antiproliferative activity against the HCT116 colon cancer and MDA-MB-231 breast cancer cell lines; and their radical scavenging activity against the 2,2′-azino-bis(3-ethylbenzothiazoline-6-sulfonic acid) (ABTS) radical cation and 2,2-diphenyl-1-picrylhydrazyl (DPPH) radical species. Four derivatives were found to have improved the antiproliferative activity compared to quercetin whilst retaining radical scavenging activity.

## 1. Introduction

**Figure 1:**
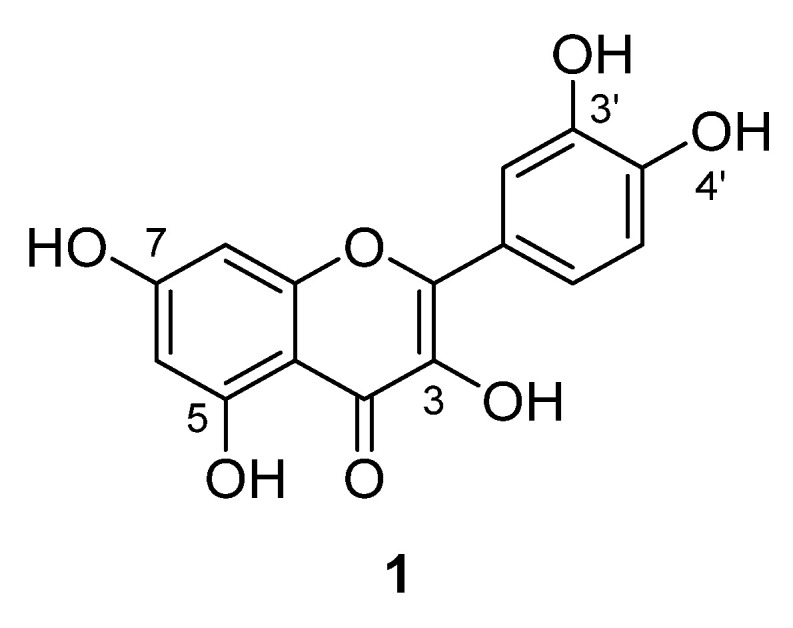
Structure of quercetin, a pentahydroxy flavonol.

Quercetin (**1**) is a flavonoid found in a wide range of commonly eaten fruits and vegetables [1,2]. Structurally, this compound belongs to the flavonol subclass of flavonoids and consists of five hydroxy groups at the 3-, 5-, 7-, 3′- and 4′-positions (Figure 1). Quercetin displays a wide range of biological activities [3], and is particularly known for its antioxidant [3,4] and antiproliferative [5,6] properties. These activities imply that quercetin have potential for the development of health-promoting agents. However, this potential is severely hindered by its poor bioavailability [7]. Previous studies have demonstrated that by increasing the lipophilicity of flavonoids this had resulted in compounds with improved biological activity [8,9,10,11,12] as well as improved properties related to bioavailability [9,13]. It was envisaged that the strategic derivatisation of quercetin could increase its lipophilicity as well as retain, or improve, its desired bioactivity. Previous studies have identified that the hydroxy groups on 3-, 3′- and 4′- positions of quercetin significantly contribute to the compound’s radical scavenging activity [14,15]. Proposedly, the mechanism of activity occurs through two proton-coupled electron transfer steps, resulting in the structural transformation from quercetin to an *ortho*-qunione which further isomerises into either of the three *para*-quinone methide structures [16]. This mechanism suggests involvement of the three hydroxy groups already mentioned, as well as that on the 7- position. Therefore, it was rationalised the 5-*O* position of quercetin would be an ideal site for lipophilic derivatisation. In this report, the syntheses of seven 5-*O* acyl quercetin derivatives are described. The derivatives were assessed on their antiproliferative activities against two cancer cell lines as well as their radical scavenging activities against two radical species. These results were compared to quercetin to determine the effect that 5-*O* acylation had on these desired biological activities.

## 2. Results and Discussion

### 2.1. Syntheses of 5-O-Acyl Quercetin Derivatives

**Scheme 1:**
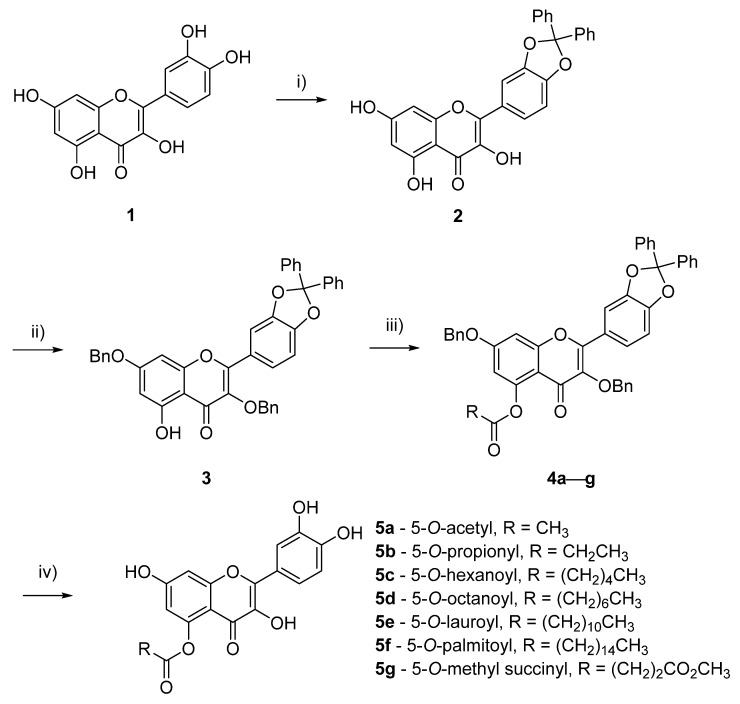
Syntheses of 5-*O*-acyl quercetin compounds: (i) Ph_2_CCl_2_, PhOPh, 175 °C, 24 h, 68%, (ii) BnBr, K_2_CO_3_, dimethylformamide (DMF), 100 °C, 24 h, 54%; (iii) Et_3_N, acyl chlorides, **4a** 75%, **4b** 94%, **4e** 88%, **4f** 88%; (iv) Pd(OH)_2_/C or Pd/C, H_2_, tetrahydrofuran (THF), r.t., 4 d, **5a** 46%, **5b** 73%, **5c** 56% over 2 steps, **5d** 60% over 2 steps, **5e** 70%, 5f 55%, **5g** 88% over two steps.

The syntheses of the 5-*O*-acyl quercetin derivatives began by protecting the catechol hydroxy groups of flavonoid **1** with a diphenyl dioxol moiety to give compound **2** (Scheme 1). Thereafter the non-hydrogen bonded hydroxy groups at the 3- and 7- positions were converted to benzyl ethers. These reactions afforded the tetra-protected quercetin **3** with an overall yield of 37%, over two steps. The experimental ^1^H NMR signals were in agreement with that previously reported in literature which gave confirmation of the tetra-protected product [17]. It was observed that the singlet at δ = 12.67, which has previously been identified as the signal for the 5-OH proton, was the only hydroxy proton signal observed to further confirm that this was the only available site for the subsequent reactions [18,19].

The addition of a range of acyl chlorides to compound **3**, in the presence of triethylamine, afforded the 5-*O*-acyl-tetra-*O*-protected derivatives (**4a**–**g**). Compounds **4a**, **4b**, **4e** and **4f** were easily separable from the starting material and were afforded in 75–94% yields. Derivatives **4c**, **4d** and **4g** were inseparable from **3** and were further reacted without purification. These penta-substituted quercetin products were immediately identified on thin layer chromatography (TLC) by their blue fluorescence under wavelength of 365 nm. Subsequently, the diphenyl dioxol and the benzyl groups of compounds **4a**–**g** were simultaneously deprotected via hydrogenation catalysed by either Pd(OH)_2_/C or Pd/C, a reaction which required 4 days. Derivatives **5a**, **5b**, **5e** and **5f** were afforded in 46–73% yield from their corresponding analogue **4**, whilst derivatives **5c**, **5d** and **5g** were afforded in 56–88% yields over two steps from compound **3**.

For all products obtained after deprotection, the four hydroxy proton signals at δ = 9.25–9.26 ppm (3-OH), δ = 11.00 (7-OH), δ = 9.49–9.53 (3′-OH) and δ = 9.25–9.28 (4′-OH) spectra were present on the ^1^H NMR spectra, whilst the proton signal for 5-OH was not observed. From ^13^C NMR spectra, it was observed that the carbon signals for C-5 of the derivatives had significantly shifted upfield, whilst the other four hydroxy bearing carbon signals remained similar, compared to that reported for quercetin [18,19]. These observations confirmed that acylation had occurred on the desired 5-*O* position.

Significant differences were observed in the chemical properties between **1** and derivatives **5a**–**g**. In contrast to their starting materials, these deprotected derivatives were easily differentiated on TLC by their yellow fluorescence under wavelength of 365 nm. The Rf values of **5a** and **5b**, acylated with the short acyl carbon chain lengths, and **5g**, with the polar methyl succinyl group, were similar to **1** (R_f_ = 0.57 in 1:2 petroleum ether: EtOAc) which suggested that these compounds have similar polarity to the parent flavonoid. Whilst, the Rf values of derivatives **5c**, **5d**, **5e** and **5f**, acylated with medium to long acyl carbon chains, suggested that they were less polar compared to quercetin. All seven derivatives **5a**–**f** were readily soluble in tetrahydrofuran solvent, whereas quercetin **1** was insoluble in this solvent. This shows that acylation at the 5-*O* position resulted in more organic soluble molecules compared to **1** alone. It was also noted that the addition of the acyl groups at the 5-*O* position had greatly reduced the melting poing of the derivatives (160–189 °C) compared that reported for **1** (313–314 °C) [20].

### 2.2. Antiproliferative Studies against HCT116 and MDA-MB-231 Cancer Cell Lines

**Figure 2:**
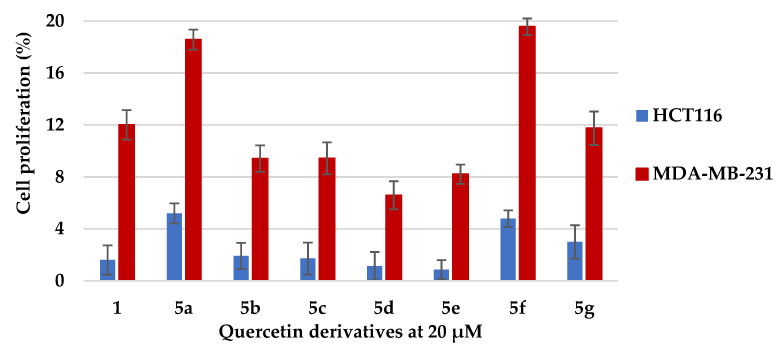
Mean cell proliferation rates (±standard error(SE) of quercetin and 5-*O*-quercetin derivatives against HCT116 and MDA-MB-231 cancer cell lines (cell proliferation in presence of control = 100%).

Previous studies have reported that quercetin **1** has antiproliferative activity against the colon HCT116 and breast MDA-MB-231 cancer cell lines and were therefore chosen to study the effect of adding the acyl groups at the 5 position [21,22,23,24]. Quercetin (**1**) and derivatives **5a**–**g** were tested against these cell lines at a single concentration of 20 μM (Figure 2). The treatment of quercetin had reduced the proliferation rates of HCT116 and MDA-MB-231 cells to 1.6 (±1.1)% and 12.0 (±1.1)% of control, respectively. In comparison, it was found that derivatives **5d** and **5e** had similar inhibitory activity against HCT116 to flavonoid **1**, with cell proliferation rates at 1.14 (±1.1)% and 0.86 (±0.7)% of control, respectively; whilst derivatives **5b**, **5c**, **5d** and **5e** had improved inhibitory activity against MDA-MB-231, with proliferation rates reduced to 9.41 (±1.0)%, 9.43 (±1.2)%, 6.59 (±1.0)% and 8.21 (±0.7)% of control, respectively.

Following this single dose study to determine activity, the IC_50_ values of these derivatives against these cell lines were then determined (Table 1). The IC_50_ of the flavonoid **1** against HCT116 and MDA-MB-231 are 5.79 (±0.13) µM and 5.81 (±0.13) µM, respectively. Derivatives **5c**–**g** displayed increased potency against both cancer cell lines, as their IC_50_ values had significantly decreased in comparison to flavonoid **1**. From these results, the **5c**, **5d**, **5e** and **5g** displayed improved activity against both cell lines compared to quercetin, with **5e** displaying the lowest IC_50_ values against HCT116 and MDA-MB-231 cell lines at 1.53 (±0.02) µM and 1.51 (±0.01) µM, respectively. The shorter acyl carbon chain derivatives **5a** and **5b** were less effective as they were observed to have reduced antiproliferative activity with higher IC_50_ values, whilst the longest acyl carbon chain derivative **5f** displayed similar activity to quercetin against both cell lines.

### 2.3. ABTS and DPPH Radical Scavenging Activity Studies

**Figure 3:**
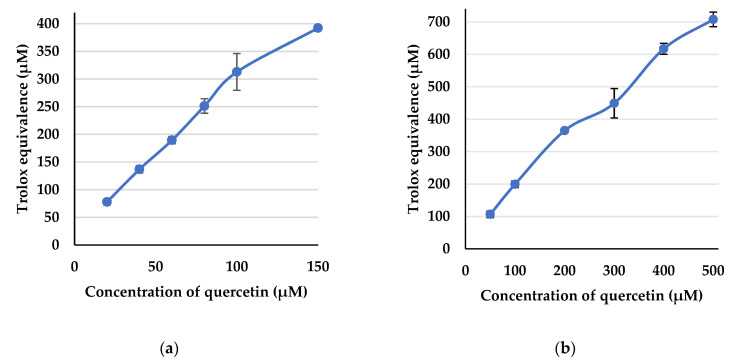
Mean Trolox equivalence (TE) concentration (±SE) of quercetin at various concentrations against (**a**) ABTS radical cation; and (**b**) against DPPH radical.

Quercetin (**1**) and derivatives **5a**–**g** were then assessed for their radical scavenging activity, and therefore their antioxidant capacity, against the two stable radical species, 2,2′-azino-bis(3-ethylbenzothiazoline-6-sulfonic acid) (ABTS) radical cation and 2,2-diphenyl-1-picrylhydrazyl (DPPH) radical. Trolox was used as a reference antioxidant. The Trolox concentration-activity standard curves were generated using 6 different concentrations of Trolox ranging between 40–400 µM against ABTS radical cation and 6 different concentrations of Trolox ranging between 80–800 µM against DPPH radical. The standard curves of Trolox against ABTS and DPPH displayed linearity of above 0.99 and 0.98, respectively (see supplementary material). The activities of quercetin against the ABTS radical cation were measured using 6 different concentrations ranging between 20–150 µM; and against the DPPH radical using 6 concentrations ranging between 50–500 µM. Using the Trolox standard curve equations, the percentage of the radical scavenged was converted to the Trolox equivalent (TE) concentration (Figure 3).

The radical scavenging activity of the quercetin derivatives **5a**–**g** was then determined (see supplementary material). These activities were compared to that of quercetin and are expressed in this report as the quercetin’s equivalence activity shown on Table 2. All derivatives, at all concentrations tested, displayed radical scavenging activity against both radicals which indicates that the acyl derivatisation at the 5-*O* position did not eliminate this important activity. It was generally found that the radical scavenging activities of the derivatives at each concentration against both radical species were not significantly different to that of quercetin (*p* > 0.05). This is with exception to **5b** (20 and 40 µM) and **5c** (20, 40, 60 and 150 µM) against ABTS as well as **5a** (200, 400 and 500 µM) and **5c** (200, 400 and 500 µM) against DPPH which were determined as significantly different (*p* < 0.05), and therefore displayed slightly reduced activity to that of quercetin.

### 2.4. General Discussion

In general, the 5-*O* acylation of quercetin improved the antiproliferative activity and did not diminish the radical scavenging activity. From antiproliferative activity studies against the two cancer cell lines, derivatives **5c**, **5d**, **5e** and **5g** had displayed lower IC_50_ values, indicating improvement, whereas derivatives **5a** and **5b**, with shorter acyl carbon chains, had reduced effect. From radical scavenging activity studies, the activities of **5d**, **5e** and **5g** against the radical species were not significantly different to that of quercetin, whilst **5a**, **5b** and **5c**, at some concentrations, did have slightly reduced activity. Therefore, derivatives **5d**, **5e** and **5g** were determined as having better, overall, activity in this study, with derivative **5e** possessing the lowest IC_50_ values against the tested cancer cell lines and without compromise towards radical scavenging activity against the two radical species.

In comparison, it was deduced that longer acyl chain derivatives of quercetin had better overall activity compared to the shorter acyl chain derivatives, suggesting that increased lipophilic character leads to increased bioavailability and improved activity. These results are in accordance with previous studies which have shown esters of flavonoids have increased bioavailability [9,13]. Though in those cases reducing radical scavenging was seen due to derivatization of the phenols known to be critical for this activity. Derivative **5g** was also effective, which suggests that the biological effects from derivatisation with polar functional groups could also be explored.

## 3. Materials and Methods

### 3.1. General Synthetic Procedures

*General Procedure A: Acylation of phenol:* To a stirred solution of phenol (1 mmol) in CH_2_Cl_2_ (25 mL) was added Et_3_N (3 mmol) and the mixture was stirred for 5 min. Acid chloride (2–5 mmol) was then added and the reaction mixture was stirred for 2–24 h. The reaction mixture was quenched by addition of saturated aqueous NaHCO_3_. This mixture was extracted with CH_2_Cl_2_ (3 × 20 mL). The combined organic extracts were dried (MgSO_4_) and the solvent removed in vacuo. The crude product was purified by flash chromatography to afford the desired product.

*General Procedure B: Removal of diphenyl dioxol and benzyl groups:* To a stirred solution of benzyl ether (1 mmol) in THF (50 mL) was added 10% Pd/C or 20% Pd(OH)_2_ (0.2 mmol) and the mixture was placed under an atmosphere of H_2_. The reaction mixture was stirred for 24 h or 4 d, as specified. This mixture was then filtered through celite, washing with THF. The solvent was removed in vacuo. The crude product was purified by flash chromatography to afford the desired product.

### 3.2. Specific Synthetic Procedures and Characterisation

*2-(2*′*,2*′*-Diphenylbenzo[d][1*′*,3*′*]dioxol-5-yl)-3,5,7-trihydroxy-4H-chromen-4-one* (**2**): To a stirred solution of quercetin **1** (3.0 g, 9.93 mmol) in diphenylether (60 mL) at 60 °C was added dichlorodiphenylmethane (2.85 mL, 14.89 mmol). The reaction mixture was then stirred at 175 °C for 24 h. The mixture was cooled to r.t. and petroleum ether (50 mL) was added to precipitate the crude product. The precipitate was filtered and further washed with petroleum ether (60 mL). The crude solid was dissolved in EtOAc and the solvent removed in vacuo. The resulting crude product was purified by flash chromatography (4:1 Petroleum ether:EtOAc) to give the title compound **2** (3.13 g, 68%) as a yellow solid. Rf: 0.30 (4:1 Petroleum ether:EtOAc). M.P.: 240–242 °C (literature 218–219 °C) [17]. δ_H_ (400 MHz; d_6_-DMSO): 6.20 (1H, d, *J* = 2.0 Hz, 6-H), 6.47 (1H, d, *J* = 2.0 Hz, 8-H), 7.22 (1H, d, *J* = 8.3 Hz, 7′-H), 7.43–7.49 (6H, m, Ar-H), 7.55–7.58 (4H, m, Ar-H), 7.80–7.83 (2H, m, 4′-H and 6′-H), 9.63 (1H, s, 3-OH), 10.82 (1H, s, 7-OH), 12.38 (1H, s, 5-OH). δ_C_ (100 MHz; d_6_-DMSO): 93.6 (C-8), 98.3 (C-6), 103.1 (C-4a), 107.8 (C-4′), 108.8 (C-7′), 117.0 (C-2′), 123.0 (C-6′), 125.2 (C-5′), 125.8 (C-2″), 128.6, 129.5 (C-3″ and C-4″), 136.4 (C-3), 139.4 (C-1″), 145.6 (C-2), 146.7 (C-3′a), 147.6 (C-7′a), 156.2 (C-8a), 160.7 (C-5), 164.1 (C-7), 176.0 (C-4). IR: νmax/cm^−1^; 696, 730, 747, 757, 775, 817, 827, 850, 871, 906, 951, 987, 1003, 1019, 1044, 1093, 1122, 1155, 1190, 1209, 1237, 1256, 1316, 1347, 1389, 1439, 1487, 1522, 1566, 1596, 1614, 1631, 1653, 2586, 3064, 3334. HRMS (ESI^+^): Found (MNa^+^) 489.0929, C_28_H_18_NaO_7_ requires 489.0945. The ^1^H NMR δ values are in agreement with literature [17].

*3,7-Bis(benzyloxy)-2-(2′,2′-diphenylbenzo[d][1′,3′]dioxol-5-yl)-5-hydroxy-4H-chromen-4-one* (**3**): To a stirred solution of **2** (2.5 g, 5.36 mmol) and K_2_CO_3_ (1.63 g, 11.79 mmol) in DMF (100 mL) at r.t. was added benzyl bromide (1.40 mL, 11.79 mmol) and the reaction mixture was stirred at 110 °C for 24 h. The reaction mixture was quenched by addition of H_2_O (70 mL). The resulting mixture was extracted with CH_2_Cl_2_ (3 × 70 mL). The combined organic extracts were further washed with excess H_2_O (3 × 70 mL). The organic extract was dried (MgSO_4_) and the solvent removed in vacuo. The crude product was purified by flash chromatography (9:1 Petroleum ether:EtOAc) to give the title compound **3** (1.88 g, 54%) as a yellow solid. Rf: 0.48 (9:1 Petroleum ether:EtOAc). M.P.: 130–132 °C (literature 90–92 °C) [17]. δ_H_ (400 MHz; CDCl_3_): 5.04 (2H, s, 3-*O*CH_2_), 5.13 (2H, s, 7-*O*CH_2_Ar), 6.44 (1H, d, *J* = 2.5 Hz, 6-H), 6.48 (1H, d, *J* = 2.5 Hz, 8-H), 6.92 (1H, d, *J* = 8.3 Hz, 7′-H), 7.14–7.22 (3H, m, Ar-H), 7.26–7.29 (2H, m, Ar-H), 7.34–7.45 (11H, m, Ar-H), 7.50 (1H, d, *J* = 2.0 Hz, 4′-H), 7.57 (1H, dd, *J* = 8.3, 2.0 Hz, 6′-H), 7.59–7.62 (4H, m, 2″-H). δ_C_ (100 MHz; CDCl_3_): 70.6 (7-*O*CH_2_), 74.6 (3-*O*CH_2_), 93.2 (C-8), 98.8 (C-6), 106.3 (C-4a), 108.5 (C-7′), 109.2 (C-4′), 124.2 (C-6′), 124.4 (C-5′), 126.4 (C-2″) 128.3, 128.5, 128.9, 129.1, 129.5 (Ar-C), 135.9 (7-*O*CH_2_C(Ar)), 136.3 (3-*O*CH_2_C(Ar)), 137.4 (C-3), 139.9 (C-1″), 147.4 (C-3′a), 149.4 (C-7′a), 156.8, 156.9 (C-2 and C-8a), 162.2 (C-5), 164.6 (C-7), 178.9 (C-4). IR: νmax/cm^−1^; 694, 750, 776, 813, 909, 948, 987, 1017, 1042, 1092, 1155, 1182, 1202, 1237, 1257, 1308, 1331, 1382, 1448, 1488, 1596, 1654, 2874, 3032. HRMS (ESI^+^): Found (MNa^+^) 669.1872, C_42_H_30_NaO_7_ requires 669.1884. The ^1^H NMR δ values are in agreement with literature [17].

*3,7-Bis(benzyloxy)-2-(2′,2′-diphenylbenzo[d][1′,3′]dioxol-5-yl)-4-oxo-4H-chromen-5-yl acetate* (**4a**): The reaction was carried out according to general procedure A with **3** (0.23 g, 0.36 mmol), Et_3_N (0.15 mL, 1.08 mmol) and acetyl chloride (0.05 mL, 0.72 mmol). The crude product was purified by flash chromatography (4:1 Petroleum ether:EtOAc) to give the title compound **4a** (0.19 g, 75%) as a white solid. Rf: 0.37 (4:1 Petroleum ether:EtOAc). M.P.: 167–170 °C. δ_H_ (400 MHz; CDCl_3_): 2.49 (3H, s, 2″-H), 5.00 (2H, s, 3-*O*CH_2_), 5.13 (2H, s, 7-*O*CH_2_), 6.69 (1H, d, *J* = 2.4 Hz, 6-H), 6.85 (1H, d, *J* = 2.4 Hz, 8-H), 6.89 (1H, d, *J* = 8.4 Hz, 7′-H), 7.07–7.18 (3H, m Ar-H), 7.21–7.23 (2H, m, Ar-H), 7.34–7.45 (12H, m, 4′-H and Ar-H), 7.48 (1H, dd, *J* = 8.4, 2.0 Hz, 6′-H), 7.57–7.63 (4H, m, 2‴-H). δ_C_ (100 MHz; CDCl_3_): 21.4 (C-2″), 70.9 (7-*O*CH_2_), 74.3 (3-*O*CH_2_), 99.7 (C-8), 108.3 (C-7′), 108.8 (C-6), 109.1 (C-4′), 111.7 (C-4a), 117.8 (C-2′), 124.0 (C-6′), 124.5 (C-5′), 126.4 (C-2‴), 127.7, 128.1, 128.1, 128.5, 128.6, 128.9, 129.1, 129.5 (Ar-C), 135.5 (7-*O*CH_2_C(Ar)), 136.5 (3-*O*CH_2_C(Ar)), 139.2 (C-3), 140.0 (C-1‴), 147.3 (C-3′a), 149.1 (C-7′a), 150.7 (C-8a), 155.2 (C-2), 157.9 (C-9), 162.4 (C-7), 169.9 (C-1″), 173.3 (C-4). IR: νmax/cm^−1^; 666, 693, 717, 733, 752, 779, 794, 823, 888, 910, 947, 981, 1019, 1044, 1078, 1151, 1184, 1207, 1234, 1260, 1293, 1335, 1366, 1398, 1443, 1496, 1596, 1625, 1763, 2923, 3032, 3062. HRMS (ESI^+^): Found (MNa^+^), 711.1966, C_44_H_32_NaO_8_ requires 711.1989.

*2-(3′,4′-Dihydroxyphenyl)-3,7-dihydroxy-4-oxo-4H-chromen-5-yl acetate* (**5a**): The reaction was carried out according to general procedure B with **4a** (0.17 g, 0.24 mmol) and 20% Pd(OH)_2_/C (34 mg, 0.05 mmol). The reaction was stirred for 4 d. The crude product was purified by flash chromatography (1:1 Petroleum ether: EtOAc) to give the title compound **5a** (38 mg, 46%) as a yellow solid. Rf: 0.48 (1:2 Petroleum ether:EtOAc). M.P.: 165–168 °C. δ_H_ (400 MHz; d_6_-DMSO): 3.32 (3H, s, 2″-H), 6.55 (1H, d, *J* = 2.1 Hz, 6-H), 6.83 (1H, d, *J* = 2.1 Hz, 8-H), 6.88 (1H, d, *J* = 8.4 Hz, 5′-H), 7.51 (1H, dd, *J* = 8.4, 1.9 Hz, 6′-H), 7.66 (1H, d, *J* = 1.9 Hz, 2′-H), 9.04 (1H, s, 3-OH), 9.25 (1H, s, 4′-OH), 9.50 (1H, s, 3′-OH), 11.02 (1H, s, 7-OH). δ_C_ (100 MHz; d_6_-DMSO): 20.9 (C-2″), 100.1 (C-8), 107.6 (C-4a), 108.1 (C-6), 114.8 (C-2′), 115.6 (C-5′), 119.5 (C-6′), 122.0 (C-1′), 137.2 (C-3), 144.1 (C-2), 145.0 (C-3′), 147.3 (C-4′), 149.8 (C-5), 156.9 (C-8a), 161.7 (C-7), 168.8 (C-1″), 170.6 (C-4). IR: νmax/cm^−1^; 678, 395, 737, 785, 809, 845, 864, 910, 937, 997, 1027, 1043, 1079, 1124, 1160, 1190, 1207, 1239, 1272, 1327, 1368, 1418, 1454, 1516, 1548, 1592, 1621, 1643, 1733, 2922, 3301. HRMS (ESI^+^): Found (MNa^+^) 367.0423, C_17_H_12_NaO_8_ requires 367.0424.

*3,7-Bis(benzyloxy)-2-(2′,2′-diphenylbenzo[d][1′,3′]dioxol-5-yl)-4-oxo-4H-chromen-5-yl propionate* (**4b**): The reaction was carried out according to general procedure A with **3** (0.22 g, 0.34 mmol), Et_3_N (0.14 mL, 1.01 mmol) and propionyl chloride (0.06 mL, 0.67 mmol). The crude product was purified by flash chromatography (4:1 Petroleum ether:EtOAc) to give the title compound **4b** (0.22 g, 94%) as white solid. Rf: 0.58 (4:1 Petroleum ether:EtOAc). M.P.: 125–128 °C. δ_H_ (400 MHz; CDCl_3_): 1.34 (3H, t, *J* = 7.5 Hz, 3″-H), 2.83 (2H, q, *J* = 7.5 Hz, ″-H), 4.99 (2H, s, 3-*O*CH_2_), 5.13 (2H, s, 7-*O*CH_2_), 6.68 (1H, d, *J* = 2.4 Hz, 6-H), 6.84 (1H, d, *J* = 2.4 Hz, 8-H), 6.89 (1H, d, *J* = 8.4 Hz, 7′-H), 7.07–7.17 (3H, m Ar-H), 7.21–7.23 (2H, m, Ar-H), 7.36–7.44 (12H, m, 4′-H, Ar-H), 7.48 (1H, dd, *J* = 8.4, 1.7 Hz, 6′-H), 7.56–7.61 (4H, m, 2‴-H). δ_C_ (100 MHz; CDCl_3_): 9.0 (C-3″), 27.8 (C-2″), 70.8 (7-*O*CH_2_), 74.3 (3-*O*CH_2_), 99.6 (C-8), 108.3 (C-7′), 108.8 (C-6), 109.1 (C-4′), 111.9 (C-4a), 117.8 (C-2′), 123.9 (C-6′), 124.6 (C-5′), 126.4 (C-2‴), 127.7, 128.1, 128.1, 128.5, 128.6, 128.9, 129.2, 129.5 (Ar-C), 135.6 (7-*O*CH_2_C(Ar)), 136.5 (3-*O*CH_2_C(Ar)), 139.2 (C-3), 140.0 (C-1‴), 147.3 (C-3′a), 149.0 (C-7′a), 150.9 (C-8a), 155.1 (C-2), 157.9 (C-9), 162.4 (C-7), 173.2 (C-1″), 173.2 (C-4). IR: νmax/cm^−1^; 667, 693, 728, 746, 779, 810, 839, 885, 914, 949, 981, 1019, 1042, 1085, 1127, 1155, 1187, 1212, 1233, 1259, 1295, 1333, 1360, 1380, 1400, 1442, 1496, 1566, 1596, 1627, 1763, 1956, 2941, 3032, 3063. HRMS (ESI^+^): Found (MNa^+^) 725.2133, C_45_H_34_NaO_8_ requires 725.2146.

*2-(3′,4′-Dihydroxyphenyl)-3,7-dihydroxy-4-oxo-4H-chromen-5-yl propionate* (**5b**): The reaction was carried out according to general procedure B with **4b** (0.21 g, 0.3 mmol) and 20% Pd(OH)2/C (42 mg, 0.06 mmol). The reaction was stirred for 4 d. The crude product was purified by flash chromatography (1:1 Petroleum ether:EtOAc) to give the title compound 5b (78 mg, 73%) as a yellow solid. Rf: 0.46 (1:2 Petroleum ether:EtOAc). M.P.: 180–183 °C. δ_H_ (400 MHz; d_6_-DMSO): 1.76 (3H, t, *J* = 7.5 Hz, 3″-H), 2.70 (2H, q, *J* = 7.5 Hz, 2″-H), 6.55 (1H, d, *J* = 2.0 Hz, 6-H), 6.82 (1H, d, *J* = 2.0 Hz, 8-H), 6.88 (1H, d, *J* = 8.7 Hz, 5′-H), 7.51 (1H, dd, *J* = 8.7, 2.0 Hz, 6′-H), 7.66 (1H, d, *J* = 2.0 Hz, 2′-H), 9.00 (1H, s, 3-OH), 9.25 (1H, s, 4′-OH), 9.49 (1H, s, 3′-OH), 11.01 (1H, s, 7-OH). δ_C_ (100 MHz; d_6_-DMSO): 8.6 (C-3″), 26.9 (C-2″), 100.1 (C-8), 107.7 (C-4a), 108.2 (C-6), 114.9 (C-2′), 115.6 (C-5′), 119.5 (C-6′), 122.0 (C-1′), 137.2 (C-3), 144.1 (C-2), 145.0 (C-3′), 147.3 (C-4′), 149.9 (C-5), 156.9 (C-8a), 161.7 (C-7), 170.7 (C-1″), 172.1 (C-4). IR: νmax/cm^−1^; 694, 726, 788, 814, 841, 874, 900, 927, 997, 1091, 1154, 1189, 1267, 1311, 1364, 1412, 1448, 1508, 1547, 1593, 1735, 2944, 3323. HRMS (ESI^+^): Found (MNa^+^) 381.0577, C_18_H_14_NaO_8_ requires 381.0581.

*2-(3′,4′-Dihydroxyphenyl)-3,7-dihydroxy-4-oxo-4H-chromen-5-yl hexanoate* (**5c**): The reaction was carried out firstly according to general procedure A with **3** (0.2 g, 0.31 mmol), Et_3_N (0.13 mL, 0.93 mmol) and hexanoyl chloride (0.09 mL, 0.62 mmol) to give a crude ester which was taken to the next step without further purification. Then, according to general procedure B using the above produced ester (0.24 g, 0.32 mmol) and 20% Pd(OH)_2_/C (45 mg, 0.06 mmol). The reaction mixture was stirred for 4 d. The crude product was purified by flash chromatography (1:1 Petroleum ether:EtOAc) to give the title compound **5c** (72 mg, 56% over 2 steps) as a yellow solid. Rf: 0.63 (1:2 Petroleum ether:EtOAc). M.P.: 160–163 °C. δ_H_ (400 MHz; d_6_-DMSO): 0.91 (3H, t, *J* = 7.1 Hz, 6″-H), 1.32–1.39 (4H, m, 4″-H and 5″-H), 1.68 (2H, p, *J* = 7.5 Hz, 3″-H), 2.66 (2H, t, *J* = 7.5 Hz, 2″-H), 6.53 (1H, d, *J* = 2.1 Hz, 6-H), 6.82 (1H, d, *J* = 2.1 Hz, 8-H), 6.88 (1H, d, *J* = 8.5 Hz, 5′-H), 7.51 (1H, dd, *J* = 8.5, 2.2 Hz, 6′-H), 7.66 (1H, d, *J* = 2.2 Hz, 2′-H), 8.98 (1H, s, 3-OH), 9.25 (1H, s, 4′-OH), 9.50 (1H, s, 3′-OH), 11.00 (1H, s, 7-OH). δ_C_ (100 MHz; d_6_-DMSO): 13.9 (C-6″), 21.9 (C-5″), 23.6 (C-3″), 30.7 (C-4″), 33.5 (C-2″), 100.1 (C-8), 107.7 (C-4a), 108.2 (C-6), 114.8 (C-2′), 115.6 (C-5′), 119.5 (C-6′), 122.0 (C-1′), 137.2 (C-3), 144.0 (C-2), 145.0 (C-3′), 147.3 (C-4′), 149.9 (C-5), 156.9 (C-8a), 161.7 (C-7), 170.7 (C-1″), 171.3 (C-4). IR: νmax/cm^−1^; 662, 692, 727, 747, 791, 822, 845, 883, 928, 991, 1105, 1140, 1187, 1214, 1249, 1277, 1312, 1379, 1415, 1446, 1509, 1518, 1536, 1584, 1624, 1722, 2854, 2924, 2956, 3319. HRMS (ESI^+^): Found (MNa^+^) 423.1034, C_21_H_20_NaO_8_ requires 423.1050.

*2-(3′,4′-Dihydroxyphenyl)-3,7-dihydroxy-4-oxo-4H-chromen-5-yl octanoate* (**5d**): The reaction was carried out firstly according to general procedure A with **3** (0.2 g, 0.31 mmol), Et_3_N (0.13 mL, 0.93 mmol) and octanoyl chloride (0.19 mL, 0.62 mmol) The product was taken into the next step without purification. Then, according to general procedure B using the above produced ester (0.24 g, 0.31 mmol) and 20% Pd(OH)_2_/C (43 mg, 0.06 mmol). The reaction was stirred for 4 d. The crude product was purified by flash chromatography (2:1 Petroleum ether:EtOAc) to give the title compound **5d** (80 mg, 60% over 2 steps) as a yellow solid. Rf: 0.28 (1:1 Petroleum ether:EtOAc). M.P.: 178–181 °C. δ_H_ (400 MHz; d_6_-DMSO): 0.88 (3H, t, *J* = 6.9 Hz, 8″-H), 1.21–1.34 (6H, broad m, 5″-H, 6″-H and 7″-H), 1.33–1.38 (2H, m, 4″-H), 1.67 (2H, p, *J* = 7.6 Hz, 3″-H), 2.66 (2H, t, *J* = 7.6 Hz, 2″-H), 6.53 (1H, d, *J* = 2.5 Hz, 6-H), 6.82 (1H, d, *J* = 2.5 Hz, 8-H), 6.88 (1H, d, *J* = 8.5 Hz, 5′-H), 7.51 (1H, dd, *J* = 8.5, 2.2 Hz, 6′-H), 7.66 (1H, d, *J* = 2.2 Hz, 2′-H), 8.98 (1H, s, 3-OH), 9.26 (1H, s, 4′-OH), 9.51 (1H, s, 3′-OH), 11.01 (1H, s, 7-OH). δ_C_ (100 MHz; d_6_-DMSO): 14.0 (C-8″), 22.1 (C-7″), 24.0 (C-3″), 28.5, 28.5 (C-4″ and C-5″), 31.2 (C-6″), 33.5 (C-2″), 100.1 (C-8), 107.8 (C-4a), 108.2 (C-6), 114.9 (C-2′), 115.6 (C-5′), 119.5 (C-6′), 122.0 (C-1′), 137.2 (C-3), 144.1 (C-2), 145.0 (C-3′), 147.3 (C-4′), 149.9 (C-5), 156.9 (C-8a), 161.7 (C-7), 170.7 (C-1″), 171.4 (C-4). IR: νmax/cm^−1^; 662, 696, 718, 727, 747, 789, 823, 843, 882, 925, 990, 1006, 1114, 1139, 1158, 1185, 1214, 1277, 1315, 1356, 1383, 1400, 1417, 1446, 1509, 1541, 1583, 1624, 1634, 1724, 2854, 2920, 2952, 3335. HRMS (ESI^+^): Found (MNa^+^) 451.1368, C_23_H_24_NaO_8_ requires 451.1363.

*3,7-Bis(benzyloxy)-2-(2′,2′-diphenylbenzo[d][1′,3′]dioxol-5-yl)-4-oxo-4H-chromen-5-yl dodecanoate* (**4e**): The reaction was carried out according to general procedure A with **3** (0.2 g, 0.31 mmol), Et_3_N (0.13 mL, 0.93 mmol) and lauroyl chloride (0.14 mL, 0.62 mmol). The crude product was purified by flash chromatography (9:1 Petroleum ether:EtOAc) to give the title compound **4e** (0.23 g, 88%) as white solid. Rf: 0.23 (9:1 Petroleum ether:EtOAc). M.P.: 75–76 °C. δ_H_ (400 MHz; CDCl_3_): 0.88 (3H, t, *J* = 6.9 Hz, 12″-H), 1.22–1.41 (14H, broad m, 5″-H, 6″-H, 7″-H, 8″-H, 9″-H, 10″-H and 11″-H), 1.43–1.50 (2H, m, 4″-H), 1.83 (2H, p, *J* = 7.6 Hz, 3″-H), 2.79 (2H, t, *J* = 7.6 Hz, 2″-H), 4.99 (2H, s, 3-*O*CH_2_), 5.13 (2H, s, 7-*O*CH_2_), 6.67 (1H, d, *J* = 2.4 Hz, 6-H), 6.84 (1H, d, *J* = 2.4 Hz, 8-H), 6.88 (1H, d, *J* = 8.5 Hz, 7′-H), 7.07–7.18 (3H, m Ar-H), 7.21–7.23 (2H, m, Ar-H), 7.34–7.44 (12H, m, 4′-H and Ar-H), 7.48 (1H, dd, *J* = 8.5, 1.8 Hz, 6′-H), 7.56–7.61 (4H, m, 2‴-H). δ_C_ (100 MHz; CDCl_3_): 14.3 (C-12″), 22.8 (C-11″), 24.7 (C-3″), 29.4, 29.5, 29.5, 29.7, 29.7, 29.8, 29.8, 31.1 (C-4″, C-5″, C-6″, C-7″, C-8″ and C-9″), 32.1 (C-10″), 34.4 (C-2″), 70.8 (7-*O*CH_2_), 74.3 (3-*O*CH_2_), 99.6 (C-8), 108.3 (C-7′), 108.9 (C-6), 109.1 (C-4′), 111.9 (C-4a), 117.8 (C-2′), 123.9 (C-6′), 124.6 (C-5′), 126.4 (C-2‴), 127.7, 128.1, 128.1, 128.5, 128.6, 128.9, 129.2, 129.5 (Ar-C), 135.6 (7-*O*CH_2_C(Ar)), 136.6 (3-*O*CH_2_C(Ar)), 139.3 (C-3), 140.0 (C-1‴), 147.3 (C-3′a), 149.0 (C-7′a), 150.9 (C-5), 155.0 (C-2), 157.9 (C-9), 162.4 (C-7), 172.6 (C-1″), 173.2 (C-4). IR: νmax/cm^−1^; 695, 749, 776, 818, 841, 908, 948, 980, 1018, 1042, 1079, 1105, 1151, 1182, 1209, 1234, 1255, 1292, 1316, 1333, 1366, 1397, 1444, 1494, 1625, 1765, 2853, 2923, 3032, 3065. HRMS (ESI^+^): Found (MNa^+^) 851.3527, C_54_H_52_NaO_8_ requires 851.3554.

*2-(3′,4′-Dihydroxyphenyl)-3,7-dihydroxy-4-oxo-4H-chromen-5-yl dodecanoate* (**5e**): The reaction was carried out according to general procedure B with **4e** with (0.22 g, 0.26 mmol) and 20% Pd(OH)_2_/C (37 mg, 0.05 mmol). The reaction was stirred for 4 d. The product was purified by flash chromatography (2:1 Petroleum ether:EtOAc) to give the title compound **5e** (90 mg, 70%) as a yellow solid. Rf: 0.39 (1:1 Petroleum ether:EtOAc). M.P.: 165–168 °C. δ_H_ (400 MHz; d_6_-DMSO): 0.85 (3H, t, *J* = 7.0 Hz, 12″-H), 1.20–1.33 (14H, broad m, 5″-H, 6″-H, 7″-H, 8″-H, 9″-H, 10″-H and 11″-H), 1.34–1.38 (2H, m, 4″-H), 1.67 (2H, p, *J* = 7.6 Hz, 3″-H), 2.66 (2H, t, *J* = 7.6 Hz, 2″-H), 6.52 (1H, d, *J* = 2.1 Hz, 6-H), 6.82 (1H, d, *J* = 2.1 Hz, 8-H), 6.87 (1H, d, *J* = 8.5 Hz, 5′-H), 7.51 (1H, dd, *J* = 8.5, 2.3 Hz, 6′-H), 7.66 (1H, d, *J* = 2.3 Hz, 2′-H), 8.96 (1H, s, 3-OH), 9.25 (1H, s, 4′-OH), 9.50 (1H, s, 3′-OH), 11.01 (1H, s, 7-OH). δ_C_ (100 MHz; d_6_-DMSO): 13.9 (C-12″), 22.1 (C-11″), 24.0 (C-3″), 28.5, 28.7, 28.8, 28.9, 29.0 (C-4″, C-5″, C-6″, C-7″, C-8″ and C-9″), 31.3 (C-10″), 33.5 (C-2″), 100.1 (C-8), 107.7 (C-4a), 108.2 (C-6), 114.8 (C-2′), 115.6 (C-5′), 119.5 (C-6′), 122.0 (C-1′), 137.2 (C-3), 144.0 (C-2), 145.0 (C-3′), 147.3 (C-4′), 149.9 (C-5), 156.9 (C-8a), 161.7 (C-7), 170.6 (C-1″), 171.3 (C-4). IR: νmax/cm^−1^; 692, 715, 750, 791, 813, 844, 883, 928, 994, 1103, 1131, 1156, 1205, 1246, 1317, 1376, 1412, 1448, 1523, 1598, 1723, 2852, 2923, 3323. HRMS (ESI^+^): Found (MNa^+^) 507.1979, C_27_H_32_NaO_8_ requires 507.1989.

*3,7-Bis(benzyloxy)-2-(2′,2′-diphenylbenzo[d][1′,3′]dioxol-5-yl)-4-oxo-4H-chromen-5-yl palmitate* (**4f**): The reaction was carried out according to general procedure A with **3** (0.5 g, 0.77 mmol), Et_3_N (0.32 mL, 2.32 mmol) and palmitoyl chloride (0.47 mL, 1.55 mmol). The crude product was purified by flash chromatography (15:1 Petroleum ether:EtOAc) give the title compound **4f** (0.60 g, 88%) as a colourless oil. Rf: 0.62 (15:1 Petroleum ether:EtOAc). δ_H_ (400 MHz; CDCl_3_): 0.88 (3H, t, *J* = 6.9 Hz, 16″-H), 1.24–1.42 (22H, broad m, 5″-H, 6″-H, 7″-H, 8″-H, 9″-H, 10″-H, 11″-H, 12″-H, 13″-H, 14″-H and 15″-H), 1.43–1.49 (2H, m, 4″-H), 1.84 (2H, p, *J* = 7.6 Hz, 3″-H), 2.79 (2H, t, *J* = 7.6 Hz, 2″-H), 4.99 (2H, s, 3-*O*CH_2_), 5.13 (2H, s, 7-*O*CH_2_), 6.67 (1H, d, *J* = 2.4 Hz, 6-H), 6.84 (1H, d, *J* = 2.4 Hz, 8-H), 6.88 (1H, d, *J* = 8.3 Hz, 7′-H), 7.07–7.17 (3H, m Ar-H), 7.21–7.23 (2H, m, Ar-H), 7.34–7.45 (12H, m, 4′-H and Ar-H), 7.48 (1H, dd, *J* = 8.3, 2.0 Hz, 6′-H), 7.58–7.61 (4H, m, 2‴-H). δ_C_ (100 MHz; CDCl_3_): 14.3 (C-16″), 22.8 (C-15″), 24.7 (C-3″), 29.4, 29.5, 29.7, 29.8, 29.8 (C-4″, C-5″, C-6″, C-7″, C-8″, C-9″, C-10″, C-11″, C-12″ and C-13″), 32.1 (C-14″), 34.4 (C-2″), 70.8 (7-*O*CH_2_), 74.3 (3-*O*CH_2_), 99.6 (C-8), 108.3 (C-7′), 108.9 (C-6), 109.1 (C-4′), 111.9 (C-4a), 117.8 (C-2′), 123.9 (C-6′), 124.6 (C-5′), 126.4 (C-2‴), 127.7, 128.1, 128.1, 128.5, 128.6, 128.9, 129.2, 129.5 (Ar-C), 135.6 (7-*O*CH_2_C(Ar)), 136.6 (3-*O*CH_2_C(Ar)), 139.3 (C-3), 140.0 (C-1‴), 147.3 (C-3′a), 149.0 (C-7′a), 150.9 (C-5), 155.0 (C-2), 157.9 (C-8a), 162.4 (C-7), 172.6 (C-1″), 173.2 (C-4). IR: νmax/cm^−1^; 695, 750, 777, 818, 841, 909, 948, 980, 1018, 1043, 1107, 1151, 1183, 1209, 1234, 1255, 1292, 1316, 1333, 1366, 1398, 1444, 1494, 1625, 1766, 2852, 2922, 3034, 3611. HRMS (ESI^+^): Found (MNa^+^) 907.4189, C_58_H_60_NaO_8_ requires 907.4180.

*2-(3′,4′-Dihydroxyphenyl)-3,7-dihydroxy-4-oxo-4H-chromen-5-yl palmitate* (**5f**): The reaction was carried out according to general procedure B with **4f** (0.26 g, 0.29 mmol) and 10% Pd/C (31 mg, 0.06 mmol). The reaction was stirred for 4 d. The crude product was purified by flash chromatography (9:1 Petroleum ether:EtOAc) to give the title compound **5f** (87 mg, 55%) as a yellow solid. Rf: 0.43 (1:1 Petroleum ether:EtOAc). M.P.: 165–168 °C. δ_H_ (400 MHz; d_6_-DMSO): 0.84 (3H, t, *J* = 6.4 Hz, 16″-H), 1.17–1.34 (22H, broad m, 5″-H, 6″-H, 7″-H, 8″-H, 9″-H, 10″-H, 11″-H, 12″-H, 13″-H, 14″-H and 15″-H), 1.35–1.37 (2H, m, 4″-H), 1.67 (2H, p, *J* = 7.6 Hz, 3″-H), 2.66 (2H, t, *J* = 7.6 Hz, 2″-H), 6.52 (1H, d, *J* = 2.2 Hz, 6-H), 6.82 (1H, d, *J* = 2.2 Hz, 8-H), 6.88 (1H, d, *J* = 8.4 Hz, 5′-H), 7.51 (1H, dd, *J* = 8.4, 2.0 Hz, 6′-H), 7.66 (1H, d, *J* = 2.0 Hz, 2′-H), 8.98 (1H, s, 3-OH), 9.28 (1H, s, 4′-OH), 9.53 (1H, s, 3′-OH), 11.02 (1H, s, 7-OH). δ_C_ (100 MHz; d_6_-DMSO): 14.0 (C-16″), 22.1 (C-15″), 24.0 (C-3″), 28.5, 28.8, 28.8, 29.1, 29.1 (C-4″, C-5″, C-6″, C-7″, C-8″, C-9″, C-10″, C-11″, C-12″ and C-13″), 31.3 (C-14″), 33.5 (C-2″), 100.1 (C-8), 107.7 (C-4a), 108.2 (C-6), 114.9 (C-2′), 115.6 (C-5′), 119.6 (C-6′), 122.0 (C-1′), 137.2 (C-3), 144.0 (C-2), 145.0 (C-3′), 147.3 (C-4′), 149.9 (C-5), 156.9 (C-8a), 161.7 (C-7), 170.7 (C-1″), 171.3 (C-4). IR: νmax/cm^−1^; 658, 697, 720, 749, 788, 819, 844, 876, 921, 935, 992, 1080, 1113, 1149, 1196, 1218, 1244, 1281, 1334, 1365, 1379, 1421, 1456, 1469, 1501, 1520, 1555, 1587, 1616, 1741, 2851, 2922, 3122, 3316, 3548. HRMS (ESI^+^): Found (MNa^+^) 563.2597, C_31_H_40_NaO_8_ requires 563.2615.

*2-(3′,4′-Dihydroxyphenyl)-3,7-dihydroxy-4-oxo-4H-chromen-5-yl methyl succinate* (**5g**): The reaction was carried out firstly according to general procedure A with **3** (0.21 g, 0.32 mmol), Et_3_N (0.14 mL, 0.97 mmol) and methyl succinyl chloride (0.04 mL, 0.65 mmol) to give a crude ester which was taken to the next step without further purification. Then, according to general procedure B using the above produced ester (0.246 g, 0.32 mmol) and 20% Pd(OH)_2_/C (45 mg, 0.06 mmol). The reaction was stirred for 4 d. The product was purified by flash chromatography (1:1 Petroleum ether:EtOAc) to give the title compound **5g** (0.12 g, 88% over 2 steps) as a yellow solid. Rf: 0.48 (2:1 Petroleum ether:EtOAc). M.P.: 186–189 °C. δ_H_ (400 MHz; d_6_-DMSO): 2.73 (2H, t, *J* = 6.9 Hz, 2″-H or 3″-H), 2.95 (2H, t, *J* = 6.9 Hz, 2″-H or 3″-H), 3.64 (2H, s, 6″-H), 6.54 (1H, d, *J* = 2.1 Hz, 6-H), 6.83 (1H, d, *J* = 2.1 Hz, 8-H), 6.88 (1H, d, *J* = 8.5 Hz, 5′-H), 7.51 (1H, dd, *J* = 8.5, 2.1 Hz, 6′-H), 7.66 (1H, d, *J* = 2.1 Hz, 2′-H), 9.01 (1H, s, 3-OH), 9.26 (1H, s, 4′-OH), 9.50 (1H, s, 3′-OH), 11.04 (1H, s, 7-OH). δ_C_ (100 MHz; d_6_-DMSO): 28.4, 29.0 (C-2″ and C-3″), 51.6 (C-6″), 100.3 (C-8), 107.6 (C-4a), 108.1 (C-6), 114.9 (C-2′), 115.6 (C-5′), 119.6 (C-6′), 122.0 (C-1′), 137.2 (C-3), 144.2 (C-2), 145.0 (C-3′), 147.4 (C-4′), 149.6 (C-5), 156.9 (C-8a), 161.7 (C-7), 170.4 (C-1″), 170.7 (C-4″), 171.3 (C-4). IR: νmax/cm^−1^; 667, 694, 722, 787, 814, 841, 881, 926, 993, 1038, 1079, 1104, 1152, 1205, 1248, 1271, 1308, 1378, 1413, 1444, 1505, 1519, 1549, 1588, 1623, 1730, 2928, 3340. HRMS (ESI^+^): Found (MNa^+^) 439.0640, C_20_H_16_NaO_10_ requires 439.0649.

### 3.3. Antiproliferative Activity Procedures

The antiproliferative studies on HCT116 and MDA-MB-231 cell lines were conducted using the [^3^H]-thymidine incorporation assay method as detailed in the report by Leung et al. [25]. Essentially, the method was conducted by seeding 3000 cells in each well of the 96 well plates, with varying concentrations of inhibitors for 3 days. [^3^H]-thymidine is added to the cells and incubated for 6 h before harvest. The cell proliferation were then analysed using Trilux/Betaplate counter showing percentage of the cells with [^3^H]-thymidine incorporated into the DNA helix. The cell lines were treated with 20 µM of each of the compounds in DMSO and the antiproliferative activity was determined as cell growth percentages relative to the 100% growth in control. The IC_50_ of the derivatives against the cell lines were then determined using concentrations of 10 µM or below.

### 3.4. Radical Scavenging Activity Procedures

*Preparation of Trolox standard and quercetin derivative solutions:* Trolox standards were prepared by dissolving in DMSO to give a 2 mM stock solution which was stored in −80 °C until use. To generate the standard curve against ABTS, the stock solution was diluted with DMSO to obtain concentrations of 40, 60, 80, 100, 200 and 400 µM. For the standard curve against DPPH, the stock solution was diluted with DMSO to obtain concentrations of 80, 100, 200, 400, 600 and 800 µM. The samples were prepared by dissolving quercetin derivatives in DMSO to make 2mM stock solution. For ABTS radical scavenging assay, the sample stock solutions were further diluted to concentrations of 20, 40, 60, 80, 100 and 150 µM. For DPPH radical scavenging assay, the sample stock solutions were then further diluted to concentrations of 50, 100, 200, 300, 400 and 500 µM.

*Preparation of ABTS radical cation solution and DPPH radical solution:* The ABTS radical cation solution was prepared according to the reported procedure [26,27]. A 20 mM acetate buffer was first prepared by adding CH_3_COONa in H_2_O and then adjusting the pH of this buffer to 4.5 with AcOH. Neutral ABTS was dissolved in this buffer solution to make a 7 mM ABTS solution. A 2.45 mM persulfate solution was prepared by dissolving K_2_S_2_O_8_ in H_2_O. The ABTS solution and persulfate solution were then added together in a 1:1 ratio. This mixture was then stored in the absence of light at r.t. for 12–16 h. The absorbance of the ABTS radical cation solution was then adjusted with MeOH to 0.700 (±0.01) at 734 nM. The DPPH radical solution was prepared using a procedure described in literature by dissolving DPPH in MeOH to make 0.1 mM, which was used for immediately [28].

*Radical scavenging assessment:* The ABTS radical cation or DPPH radical solution (200 µL) were added to each well in the 96 well plate. The Trolox standard and quercetin samples (10 µL) were then added to the ABTS or DPPH solution. For control, DMSO (10 µL) was added to the well. This was then incubated in the absence of light for 1 h. The resulting absorbance (abs) was measured at 734 nm and 517 nm for ABTS and DPPH, respectively. The radical scavenging activity of the standards and samples were measured in triplicate within each experiment. The radical scavenging experiments were conducted three times using freshly prepared radical solutions and samples for each experiment. The percentage radical scavenging activity of Trolox standard against both ABTS and DPPH were calculated according to Equation (1).

% radical scavenging activity = [(abs of control − abs of sample)/abs of control] × 100(1)

Statistical analysis to compare the radical scavenging activity of each derivative at each concentration to that of quercetin was conducted using a two-sample t-test (two tailed) with unequal variances with significance level of 0.05. A *p*-value greater than 0.05 was determined as not significantly different and *p*-value equal to or less than 0.05 was determined as significantly different.

## 4. Conclusions

In conclusion, seven 5-*O*-acyl quercetin derivatives (**5a**–**g**) were synthesised and assessed on their antiproliferative activity and radical scavenging activity. In general, quercetin derivatives with longer 5-*O*-acyl carbon chain lengths displayed better activity against the HCT116 and MDA-MB-231 cancer cell lines compared to derivatives with shorter acyl chain lengths. Similarly, derivatives with longer 5-*O*-acyl carbon chain lengths had comparable radical scavenging activity against ABTS and DPPH radicals compared to quercetin, whilst derivatives with shorter acyl carbon chain lengths had slightly reduced activity. Amongst these derivatives prepared, the 5-*O*-octanoyl (**5d**), 5-*O*-lauroyl (**5e**) and 5-*O*-methyl succinyl (**5g**) quercetin derivatives displayed the best overall activity. These results show that the derivatisation of the 5-OH group can be used to modify the physical properties of quercetin whilst still maintaining, or even improving, the antiproliferative activity.

## Figures and Tables

**Table 1:** Mean IC_50_ values (±SE) of quercetin and quercetin 5-*O*-acyl quercetin derivatives.

Compound	IC_50_ (µM)	
	HCT116	MDA-MB-231
**1**	5.79 (±0.24)	5.81 (±0.13)
**5a**	7.81 (±0.85)	6.43 (±0.76)
**5b**	>10	9.91 (±0.81)
**5c**	4.29 (±0.44)	3.60 (±0.58)
**5d**	2.47 (±0.20)	2.65 (±0.06)
**5e**	1.53 (±0.02)	1.51 (±0.01)
**5f**	4.98 (±0.6)	5.02 (±0.35)
**5g**	3.36 (±0.00)	2.82 (±0.05)

**Table 2:** Quercetin’s equivalence radical scavenging activity of the 5-*O*-acyl quercetin derivatives against ABTS radical cation and DPPH radical at various concentrations.

Compound	Sample Concentration						
	ABTS DPPH	20 µM 50 µM	40 µM 100 µM	60 µM 200 µM	80 µM 300 µM	100 µM 400 µM	150 µM 500 µM
**1**	ABTS	1.00	1.00	1.00	1.00	1.00	1.00
	DPPH						
**5a**	ABTS	0.85	0.91	0.99	0.96	0.93	1.00
	DPPH	0.75	0.82	0.81	1.00	0.91	0.91
**5b**	ABTS	0.80	0.88	0.94	0.91	0.89	0.97
	DPPH	0.89	0.92	0.90	1.04	0.93	0.95
**5c**	ABTS	0.70	0.81	0.86	0.84	0.84	0.89
	DPPH	1.00	0.90	0.78	0.88	0.80	0.82
**5d**	ABTS	0.97	1.02	1.10	1.04	0.99	1.06
	DPPH	1.37	1.15	1.00	1.16	1.01	1.00
**5e**	ABTS	1.03	0.96	0.98	0.95	0.95	0.97
	DPPH	1.47	1.14	1.02	1.08	0.97	0.95
**5f**	ABTS	0.89	0.90	0.94	0.93	0.88	0.98
	DPPH	0.99	0.89	0.92	1.05	0.94	0.98
**5g**	ABTS	0.85	0.97	0.98	0.93	0.95	1.00
	DPPH	1.13	1.05	0.98	1.10	0.97	0.99

## Supplementary Materials

The ^1^H and ^13^C NMR spectra of compounds **4a**, **4b**, **4e**, **4f**, **5a**, **5b**, **5c**, **5d**, **5e**, **5f** and **5g.** Trolox standard curves (Figures S1 and S2) and the radical scavenging activity data of the quercetin derivatives (Figures S3 and S4).

## Sample Availability

Samples of the compounds are available from the authors.
