# Supplementary material for: Synthesis, Antiproliferative Activity and Radical Scavenging Ability of 5-O-Acyl Derivatives of Quercetin
Source: Molecules. 2021 Mar 14;26(6):1608. doi: 10.3390/molecules26061608 (PMC7999741; doi:10.3390/molecules26061608)
Supplement: Supplementary file 1 [file molecules-26-01608-s001.pdf]

Synthesis, antiproliferative activity and radical scavenging ability of 5-O-acyl derivatives of quercetin

Stephen Lo[1], Euphemia Leung[2], Bruno Fedrizzi[1] and David Barker[1,3*]

[1]*School of Chemical Sciences, University of Auckland, 23 Symonds St, Auckland, New Zealand*

[2]*Auckland Cancer Society Research Centre, University of Auckland, Auckland, New Zealand*

[3]*MacDiarmid Institute for Advanced Materials and Nanotechnology, Wellington, New Zealand.*

* *Correspondence: d.barker@auckland.ac.nz; Tel.: (+64 9 923 9703)*

Supplementary Material

Table of Contents:

^{1}H and ^{13}C NMR spectra 2

3,7-Bis(benzyloxy)-2-(2',2'-diphenylbenzo[d][1',3']dioxol-5-yl)-4-oxo-4*H*-chromen-5-yl acetate (4a) 2

2-(3',4'-Dihydroxyphenyl)-3,7-dihydroxy-4-oxo-4H-chromen-5-yl acetate (5a) 3

3,7-Bis(benzyloxy)-2-(2',2'-diphenylbenzo[d][1',3']dioxol-5-yl)-4-oxo-4*H*-chromen-5-yl propionate (4b) 4

2-(3',4'-Dihydroxyphenyl)-3,7-dihydroxy-4-oxo-4H-chromen-5-yl propionate (5b) 5

2-(3',4'-Dihydroxyphenyl)-3,7-dihydroxy-4-oxo-4H-chromen-5-yl hexanoate (5c) 6

2-(3',4'-Dihydroxyphenyl)-3,7-dihydroxy-4-oxo-4H-chromen-5-yl octanoate (5d) 7

3,7-Bis(benzyloxy)-2-(2',2'-diphenylbenzo[d][1',3']dioxol-5-yl)-4-oxo-4*H*-chromen-5-yl dodecanoate (4e) 8

2-(3',4'-Dihydroxyphenyl)-3,7-dihydroxy-4-oxo-4H-chromen-5-yl dodecanoate (5e) 9

3,7-Bis(benzyloxy)-2-(2',2'-diphenylbenzo[d][1',3']dioxol-5-yl)-4-oxo-4*H*-chromen-5-yl palmitate (4f) 10

2-(3',4'-Dihydroxyphenyl)-3,7-dihydroxy-4-oxo-4H-chromen-5-yl palmitate (5f) 11

2-(3',4'-Dihydroxyphenyl)-3,7-dihydroxy-4-oxo-4H-chromen-5-yl methyl succinate (5g) 12

Radical scavenging activity data 13

Trolox standard curves against ABTS 13

Trolox standard curves against DPPH 14

Radical scavenging activity of quercetin and quercetin derivatives against ABTS 15

Radical scavenging activity of quercetin and quercetin derivatives against DPPH 17

^{1}H and ^{13}C NMR spectra

3,7-Bis(benzyloxy)-2-(2',2'-diphenylbenzo[d][1',3']dioxol-5-yl)-4-oxo-4*H*-chromen-5-yl acetate (4a)

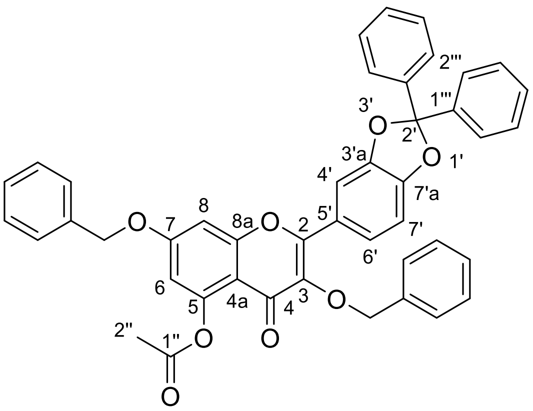

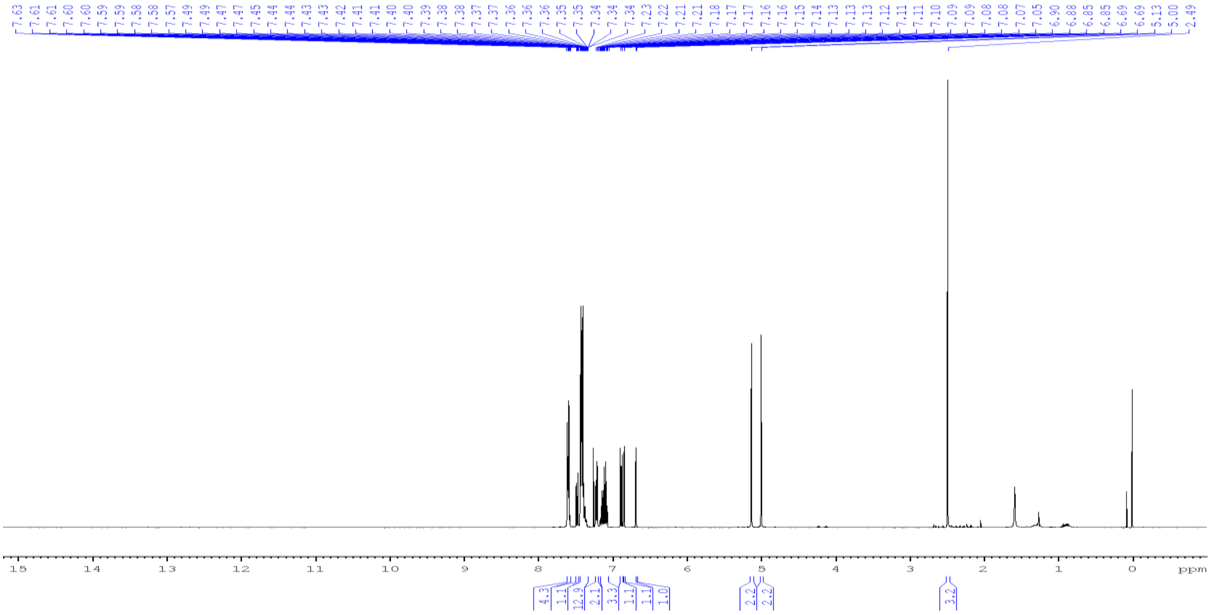

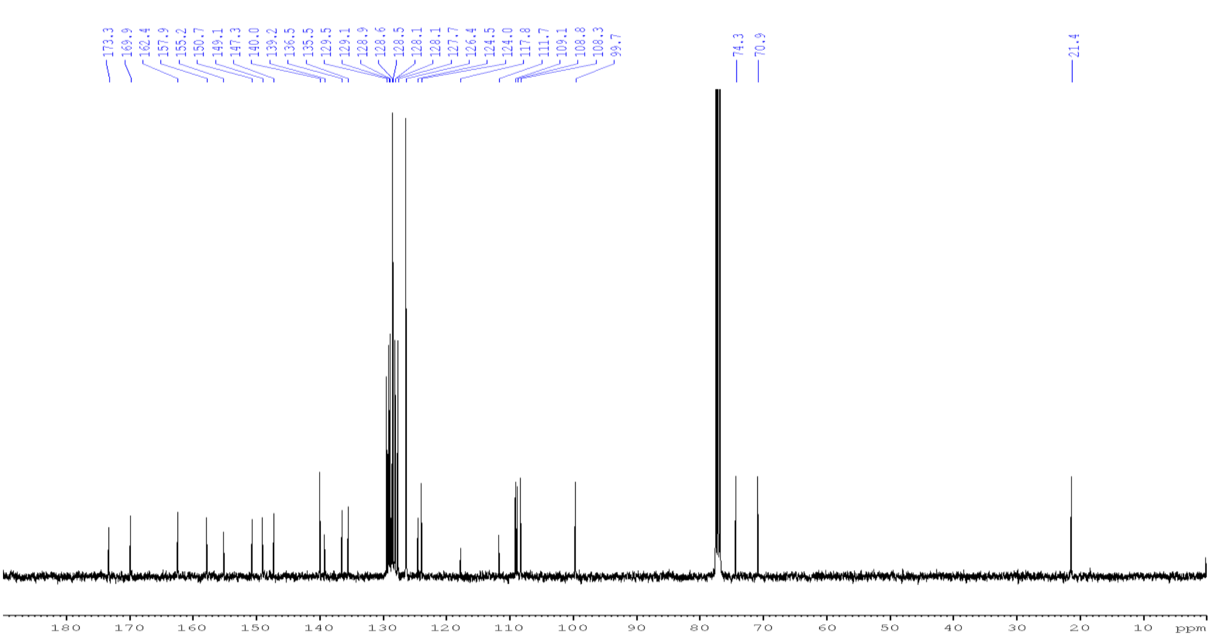

2-(3',4'-Dihydroxyphenyl)-3,7-dihydroxy-4-oxo-4H-chromen-5-yl acetate (5a)

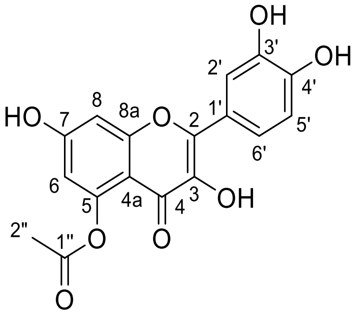

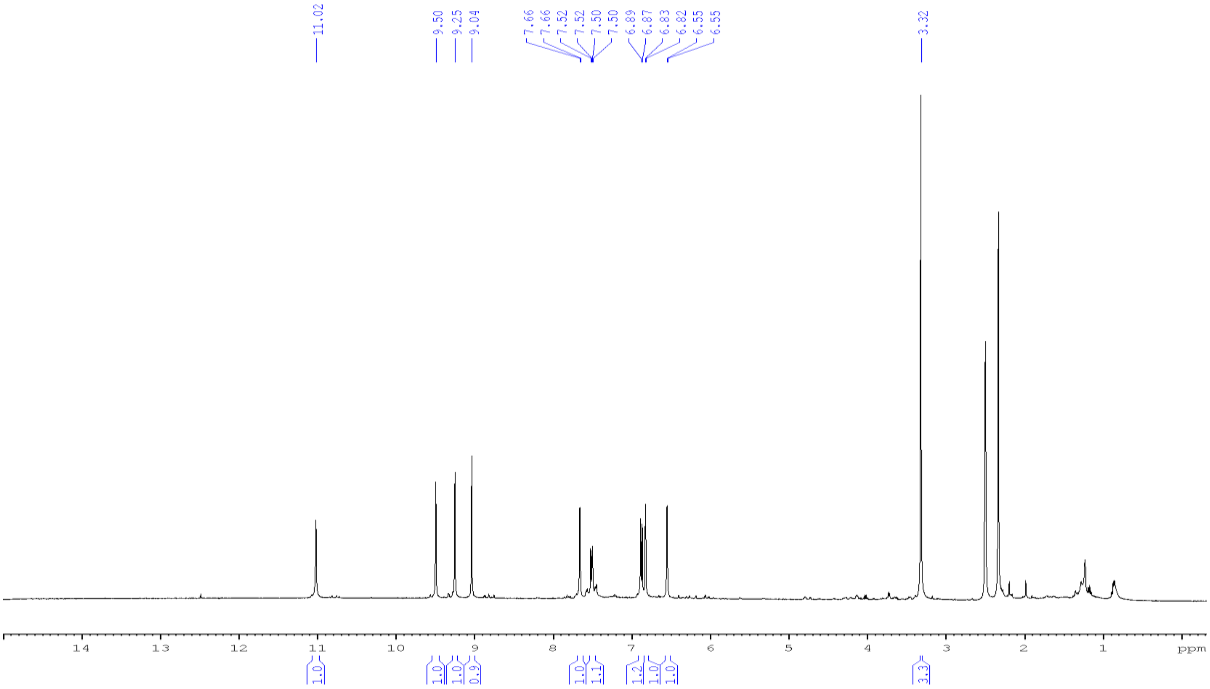

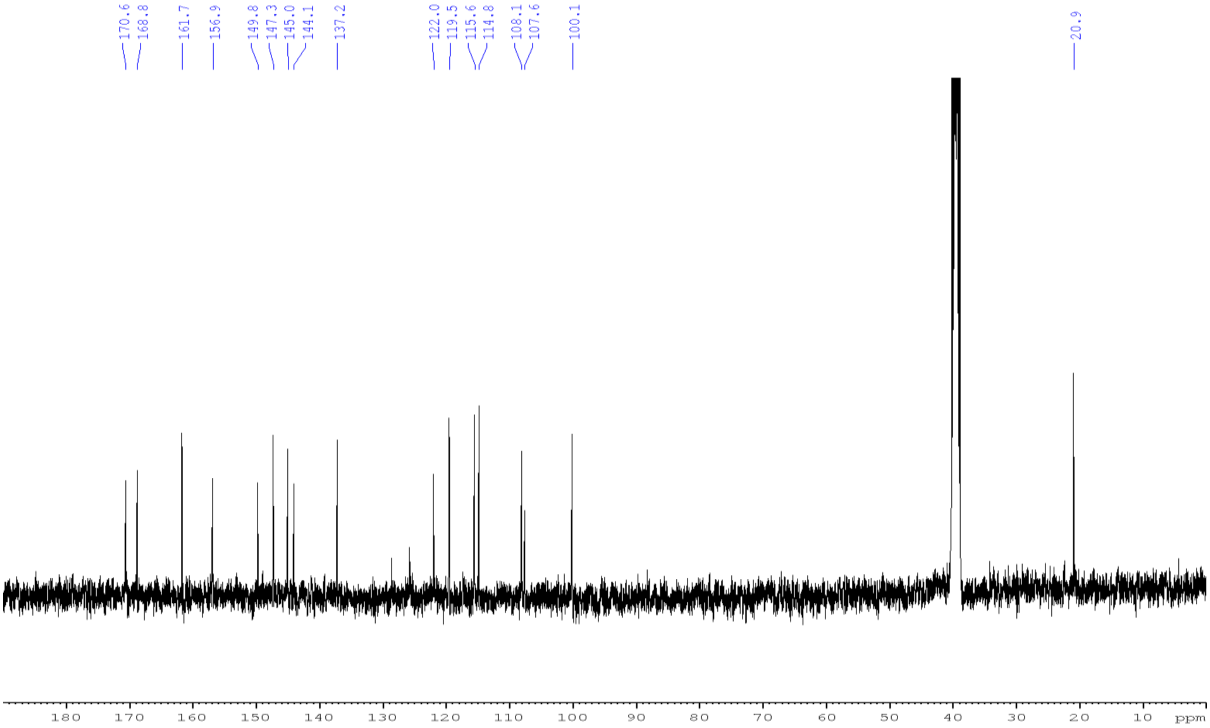

3,7-Bis(benzyloxy)-2-(2',2'-diphenylbenzo[d][1',3']dioxol-5-yl)-4-oxo-4*H*-chromen-5-yl propionate (4b)

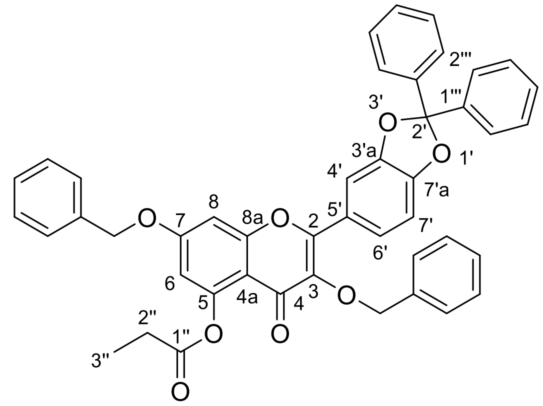

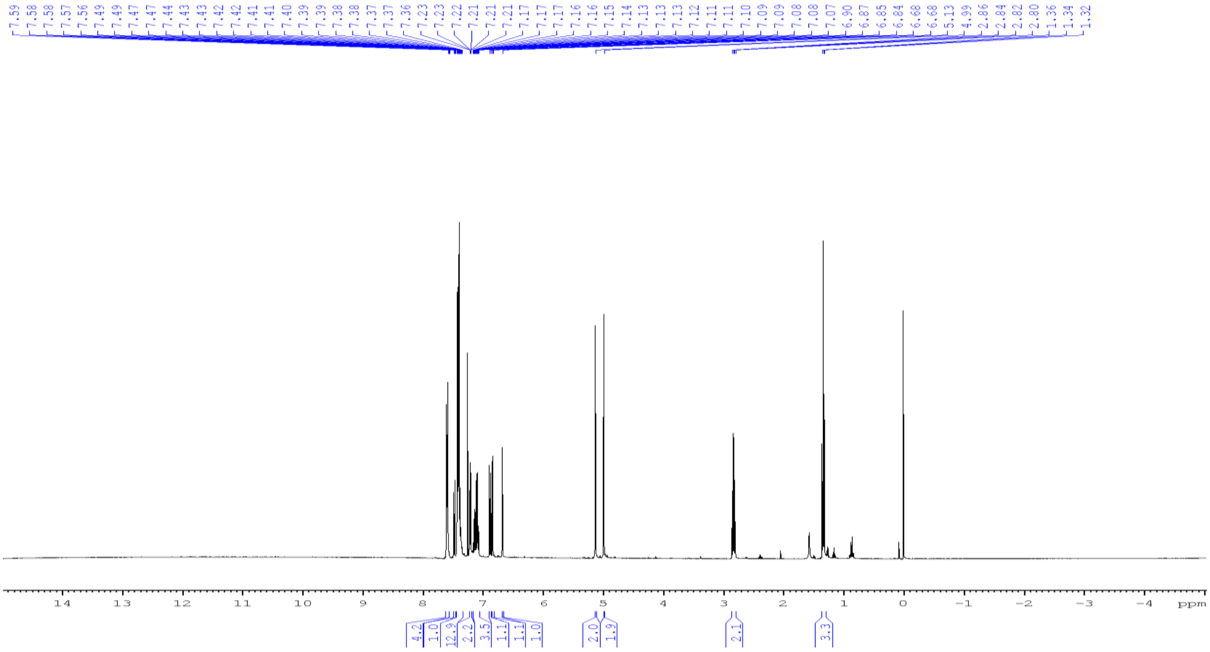

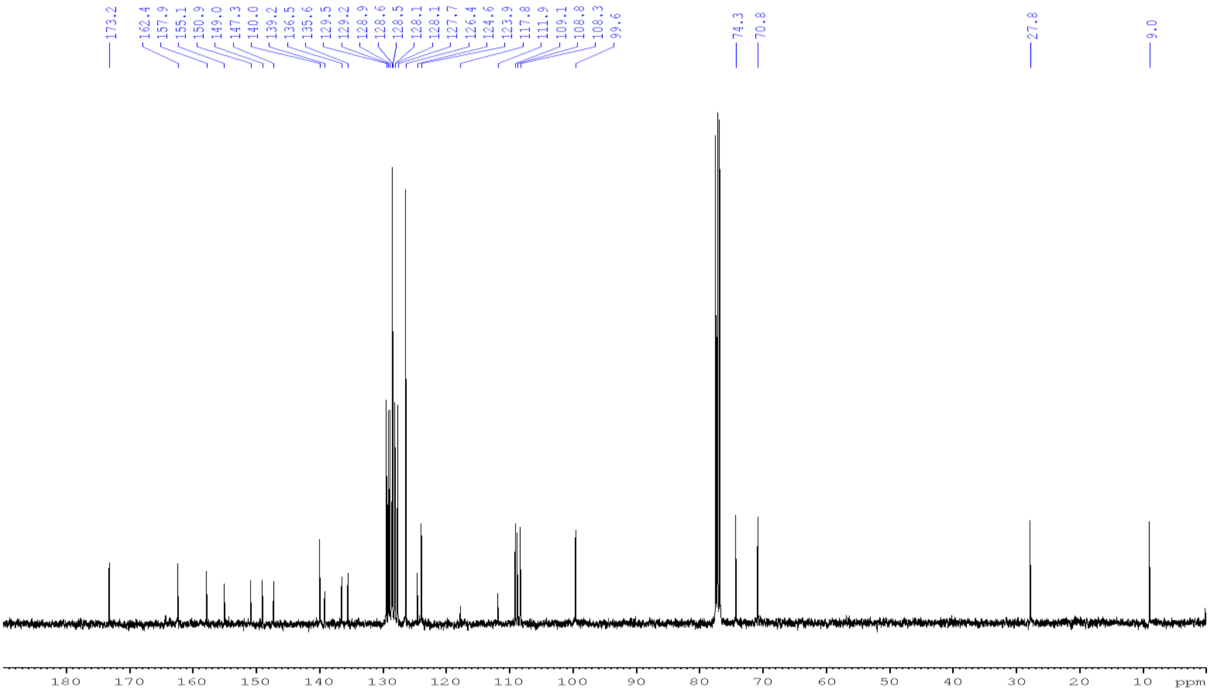

2-(3',4'-Dihydroxyphenyl)-3,7-dihydroxy-4-oxo-4H-chromen-5-yl propionate (5b)

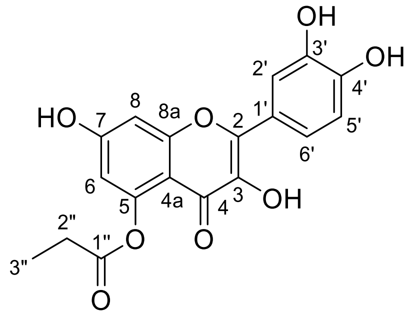

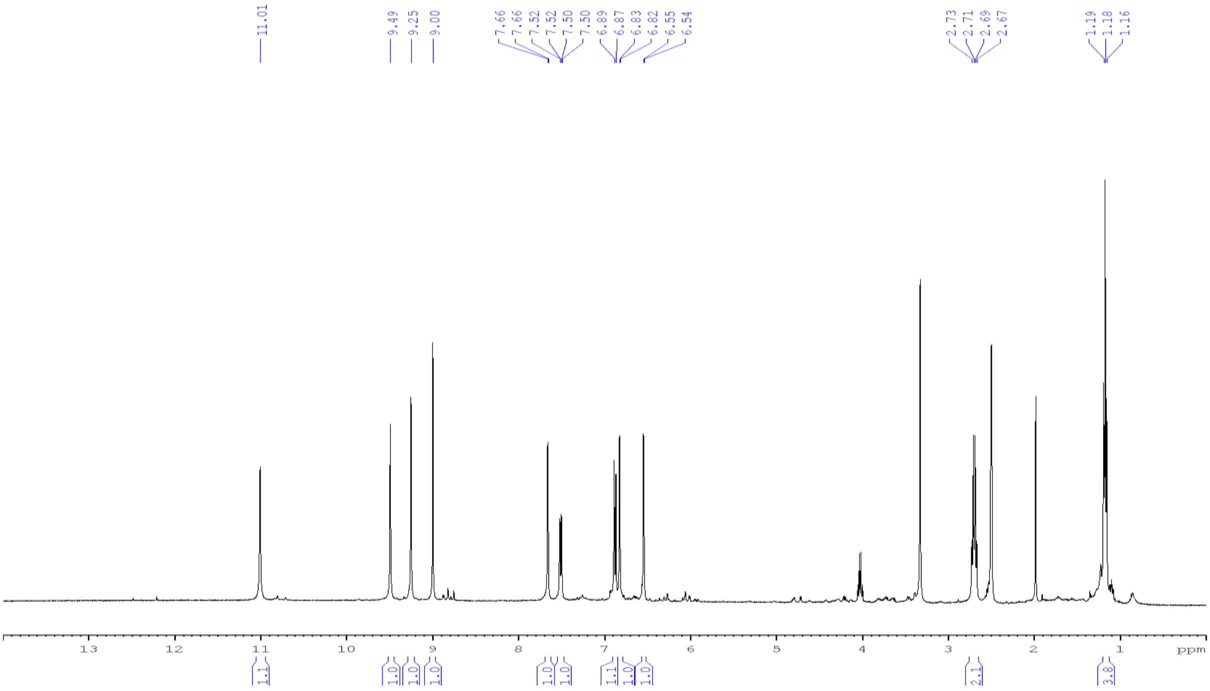

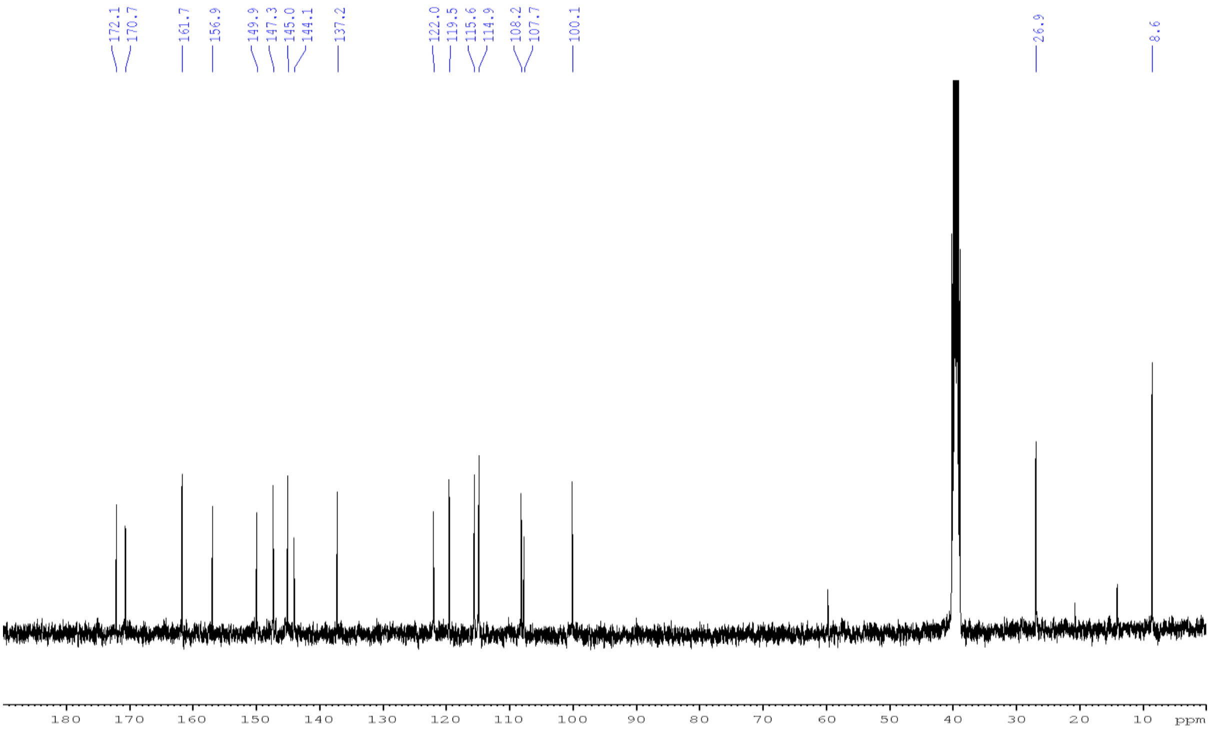

2-(3',4'-Dihydroxyphenyl)-3,7-dihydroxy-4-oxo-4H-chromen-5-yl hexanoate (5c)

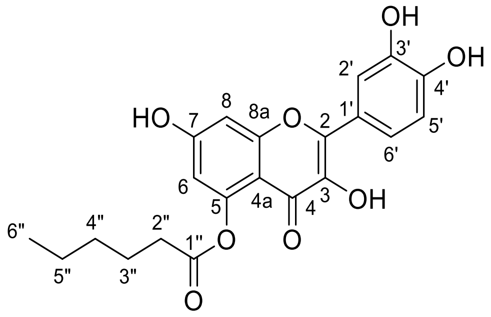

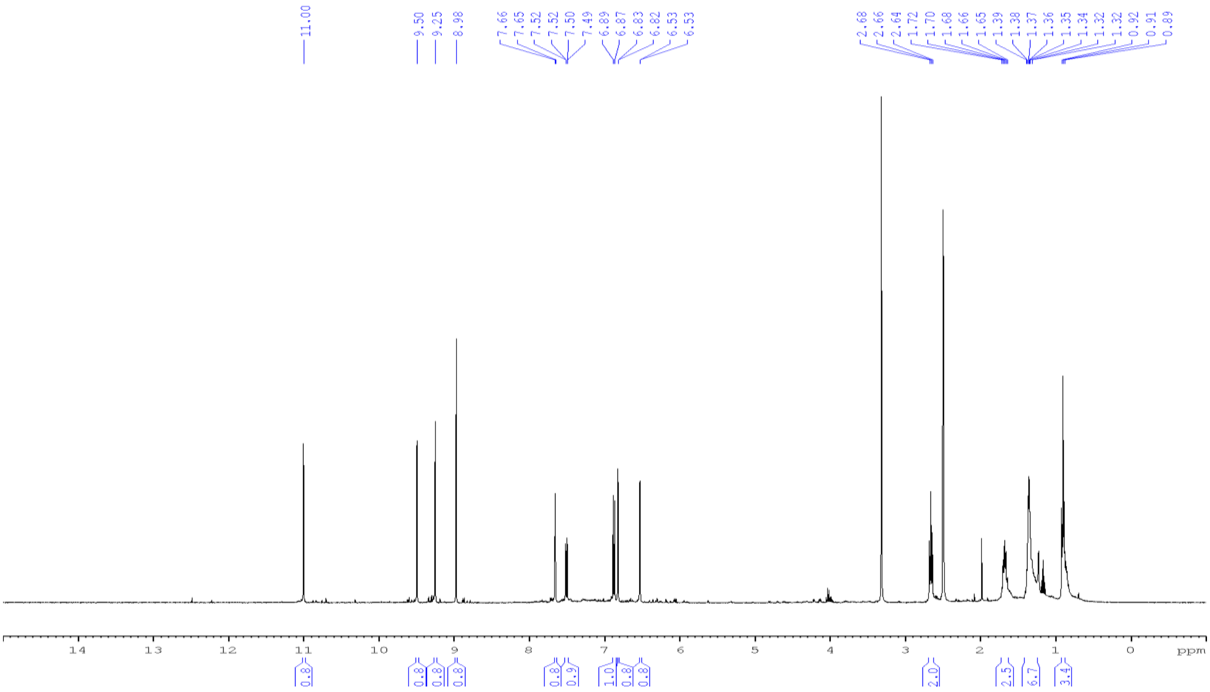

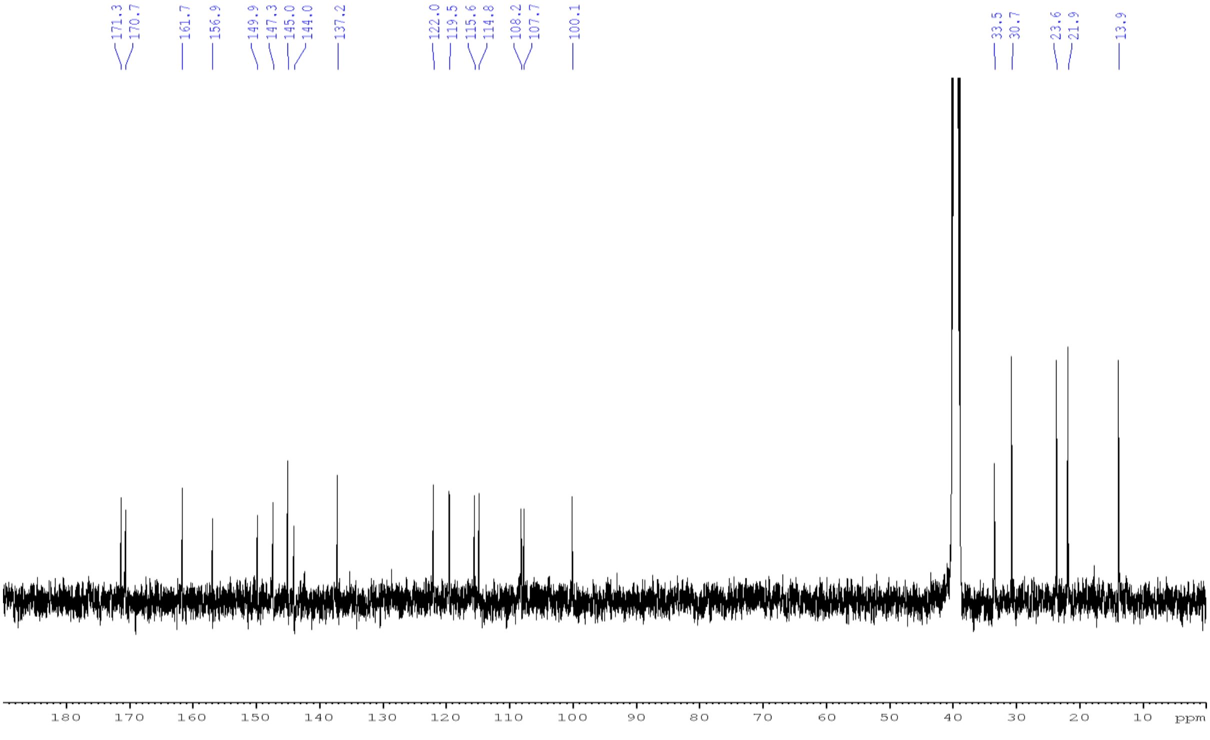

2-(3',4'-Dihydroxyphenyl)-3,7-dihydroxy-4-oxo-4H-chromen-5-yl octanoate (5d)

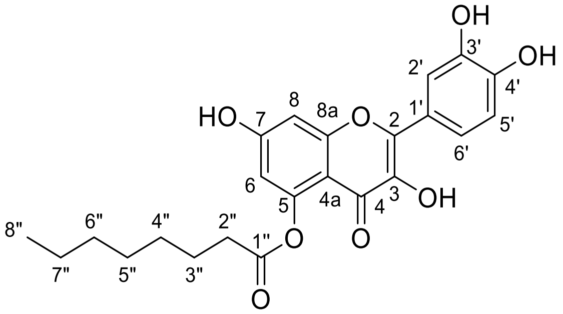

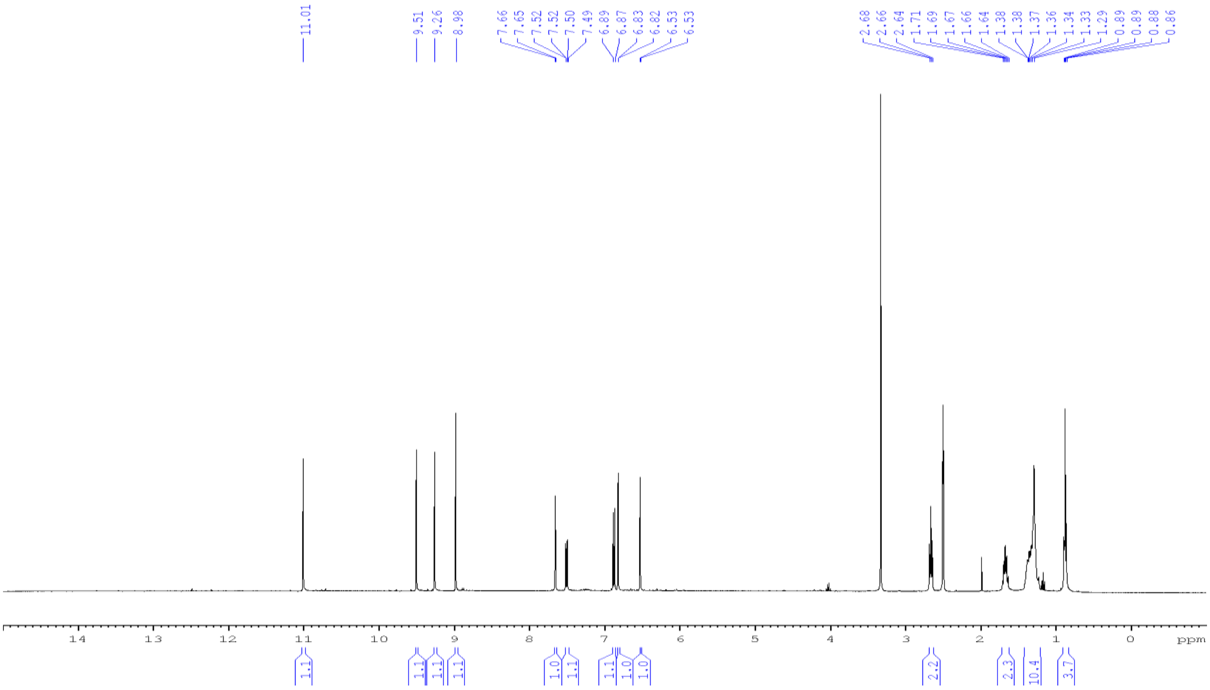

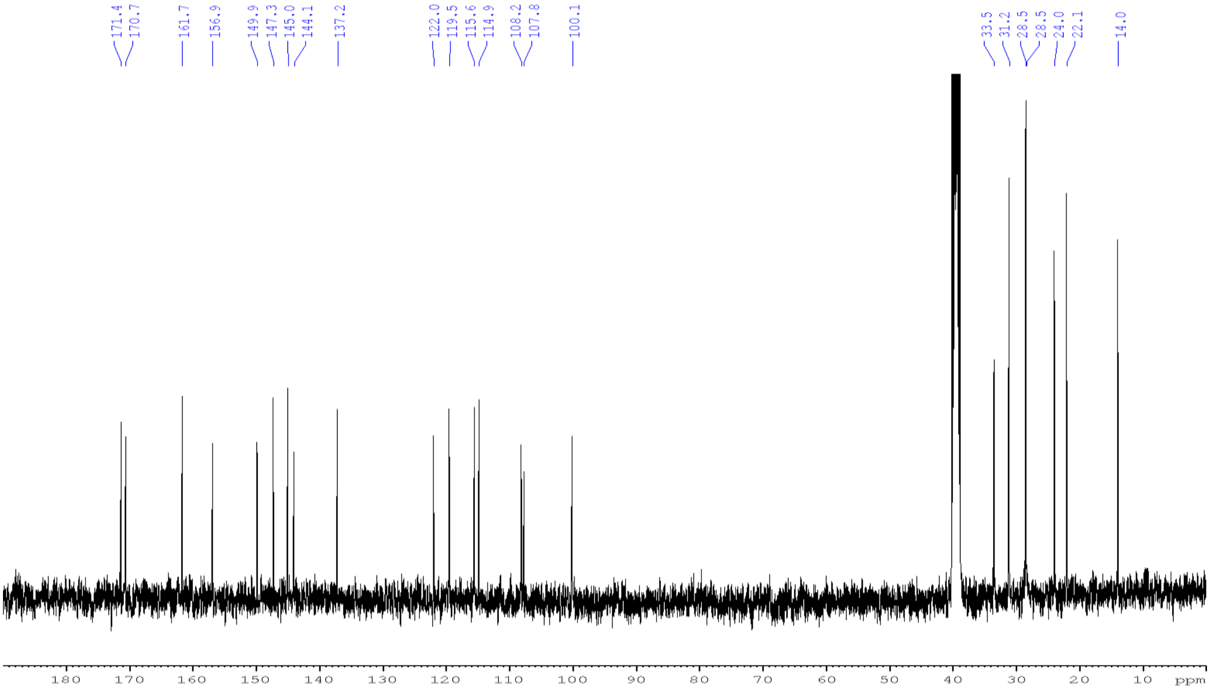

3,7-Bis(benzyloxy)-2-(2',2'-diphenylbenzo[d][1',3']dioxol-5-yl)-4-oxo-4*H*-chromen-5-yl dodecanoate (4e)

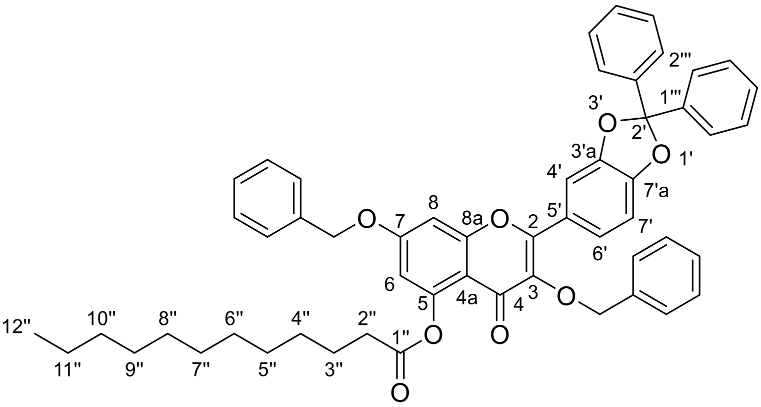

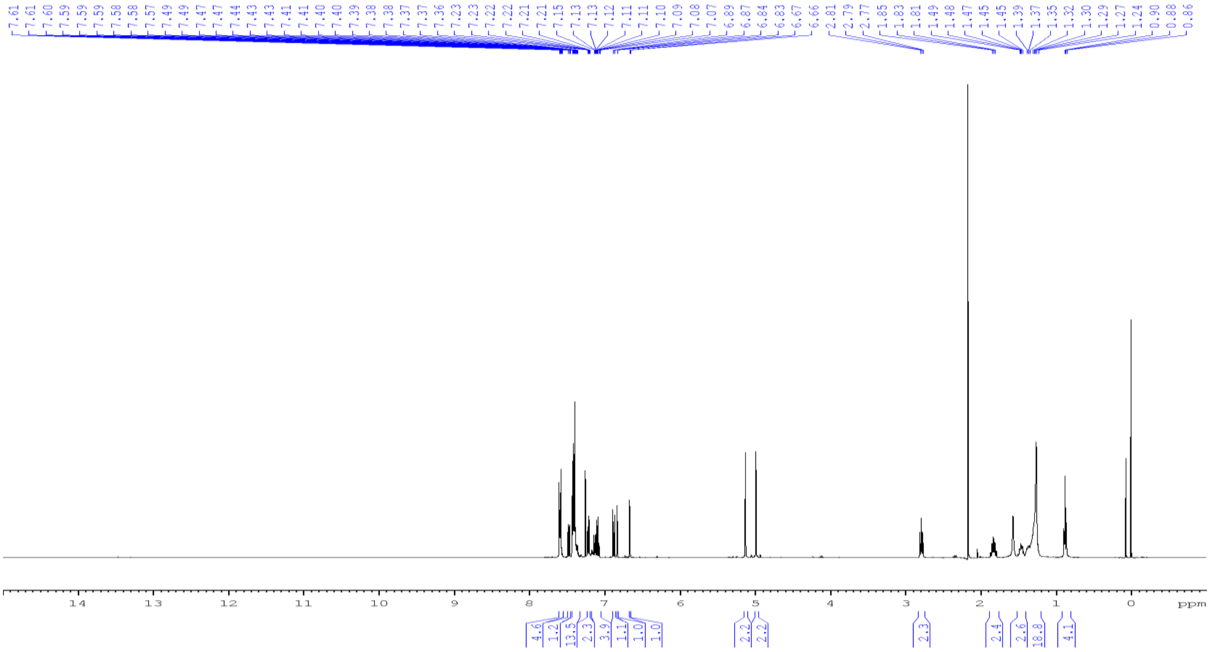

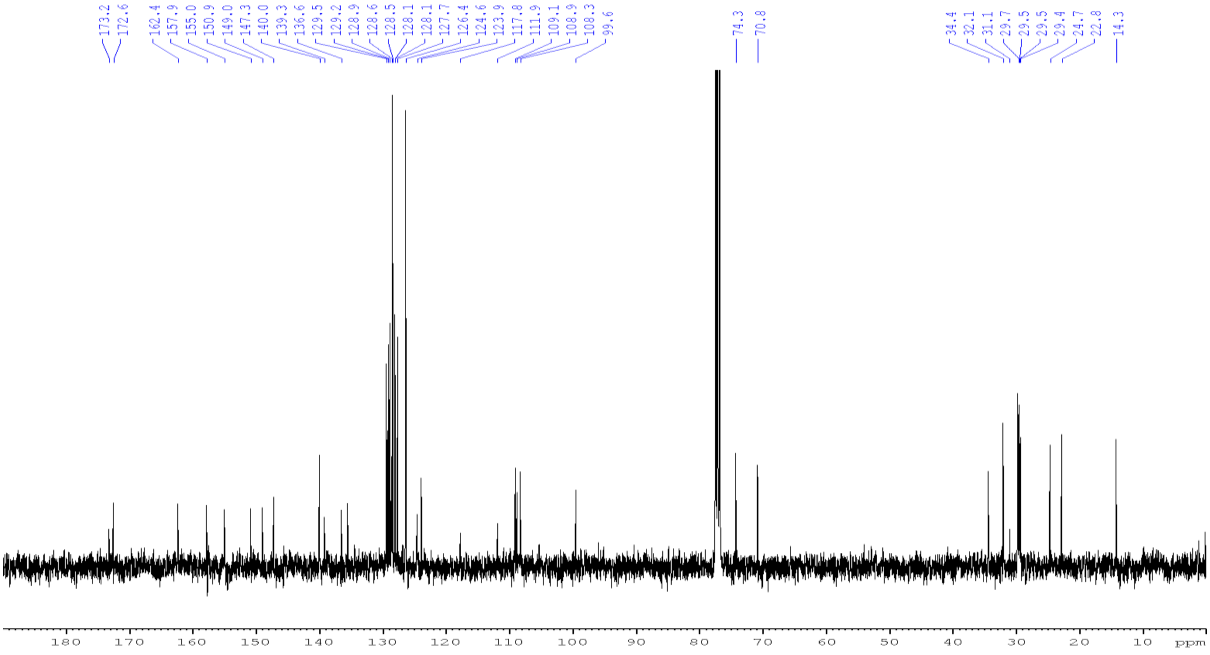

2-(3',4'-Dihydroxyphenyl)-3,7-dihydroxy-4-oxo-4H-chromen-5-yl dodecanoate (5e)

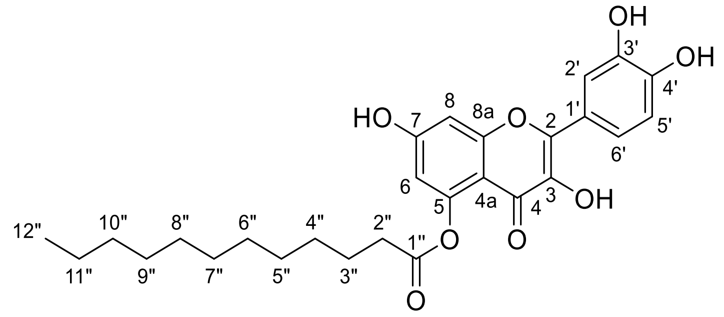

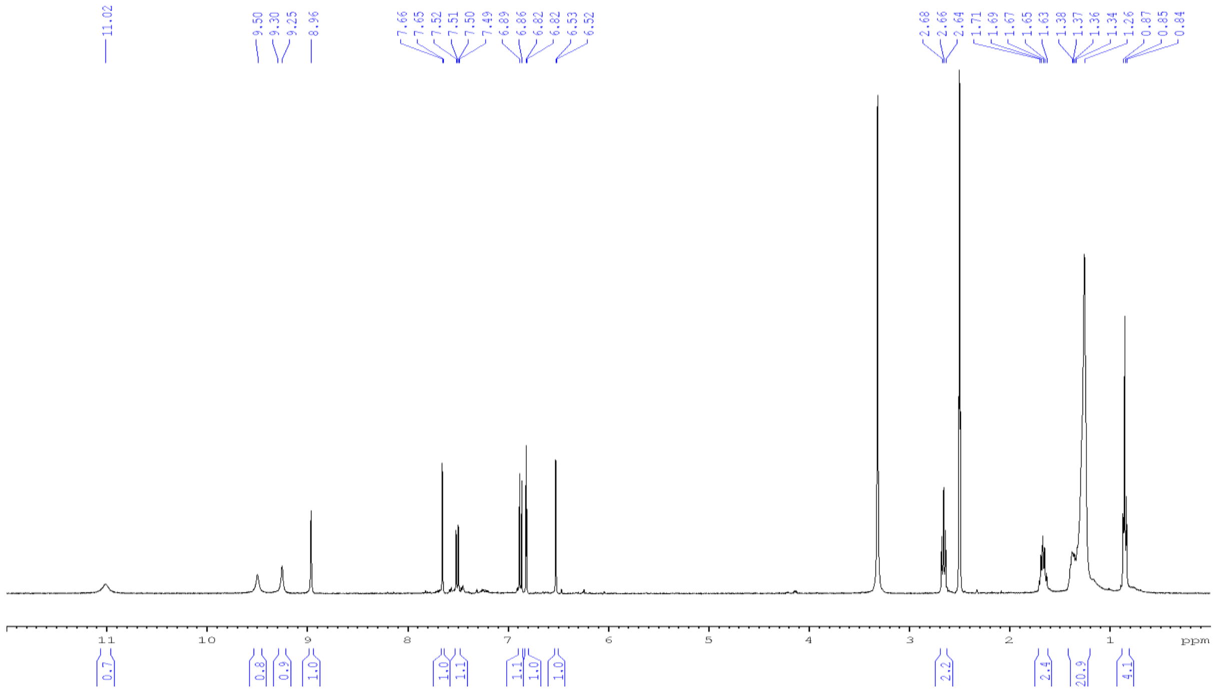

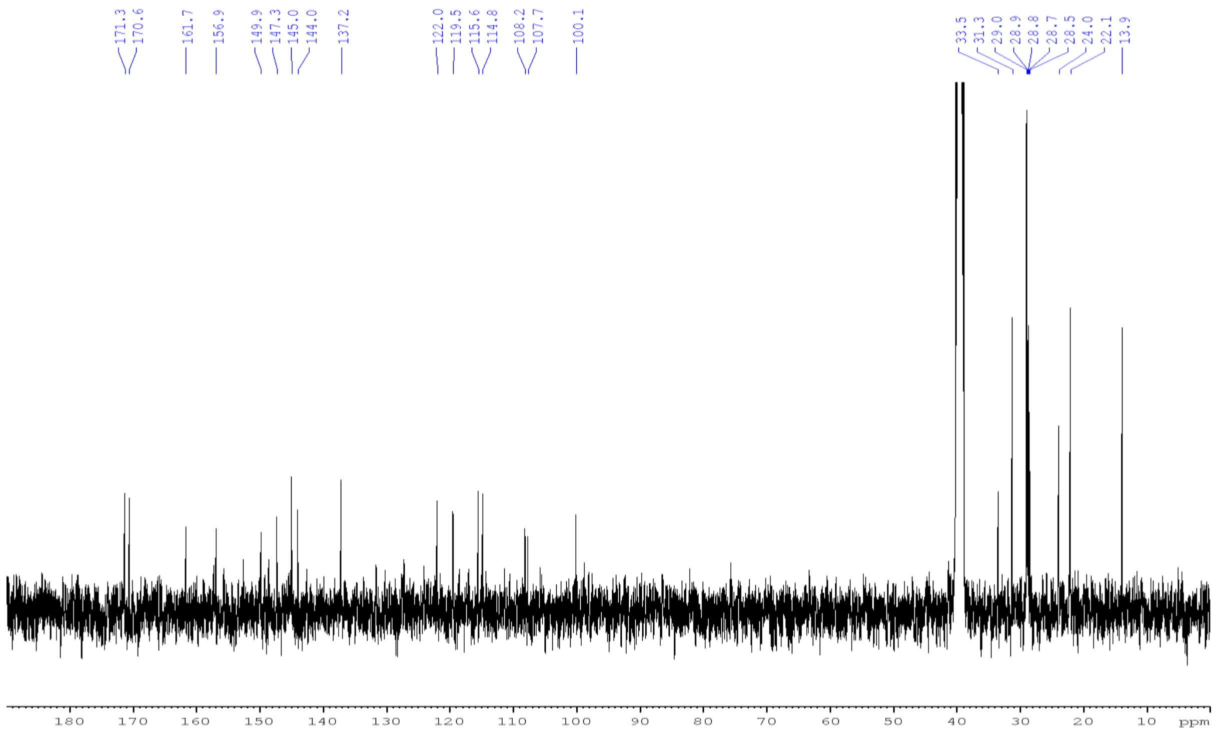

3,7-Bis(benzyloxy)-2-(2',2'-diphenylbenzo[d][1',3']dioxol-5-yl)-4-oxo-4*H*-chromen-5-yl palmitate (4f)

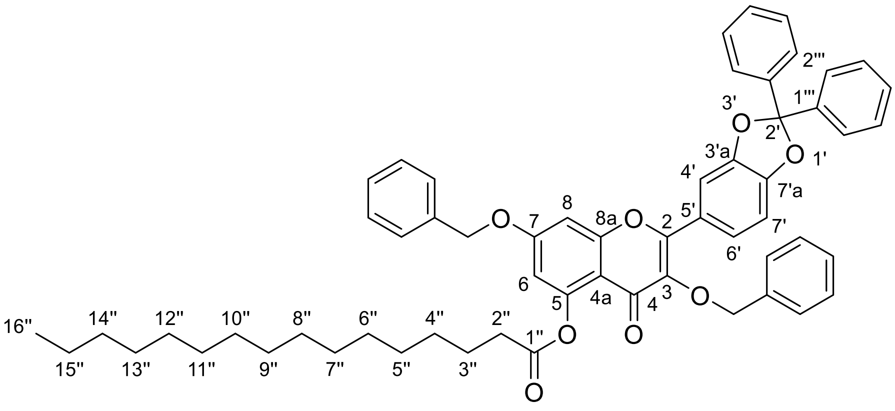

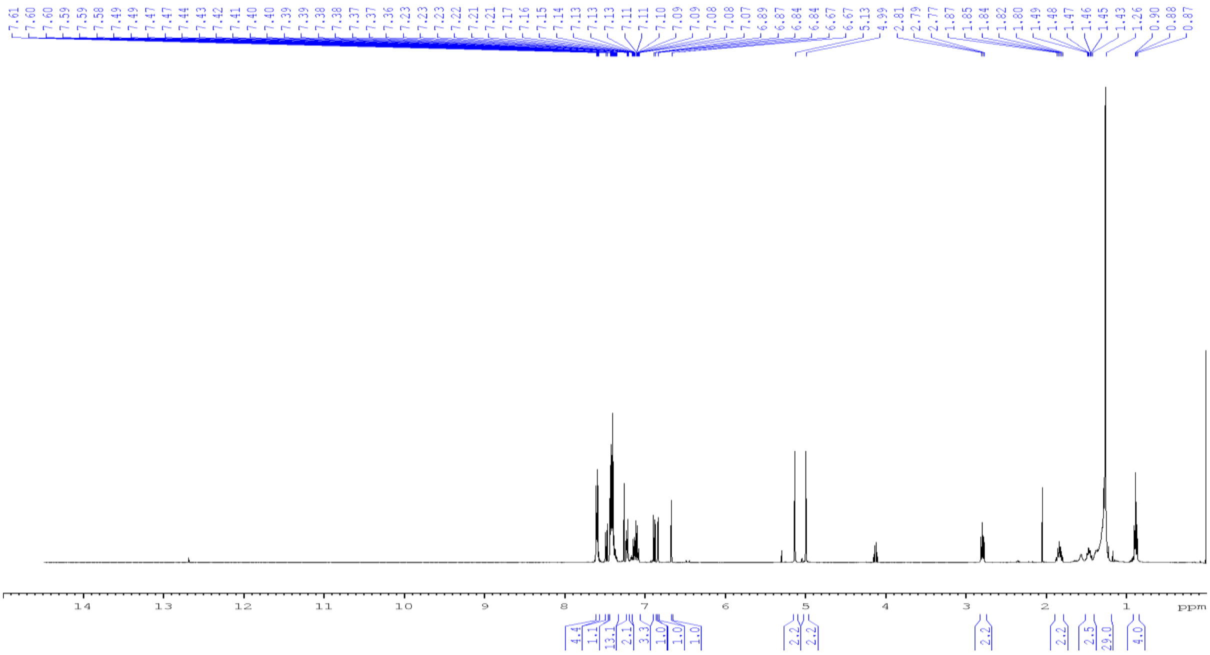

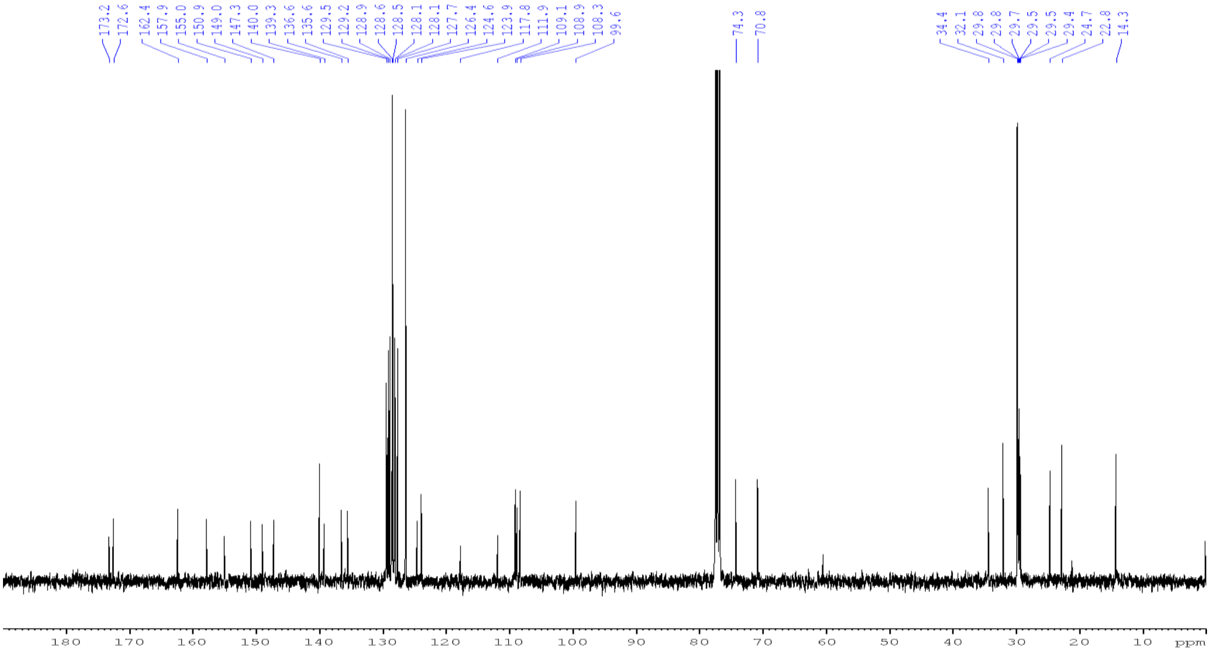

2-(3',4'-Dihydroxyphenyl)-3,7-dihydroxy-4-oxo-4H-chromen-5-yl palmitate (5f)

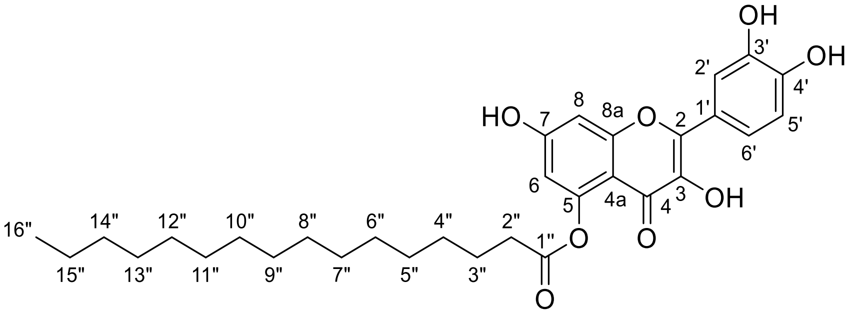

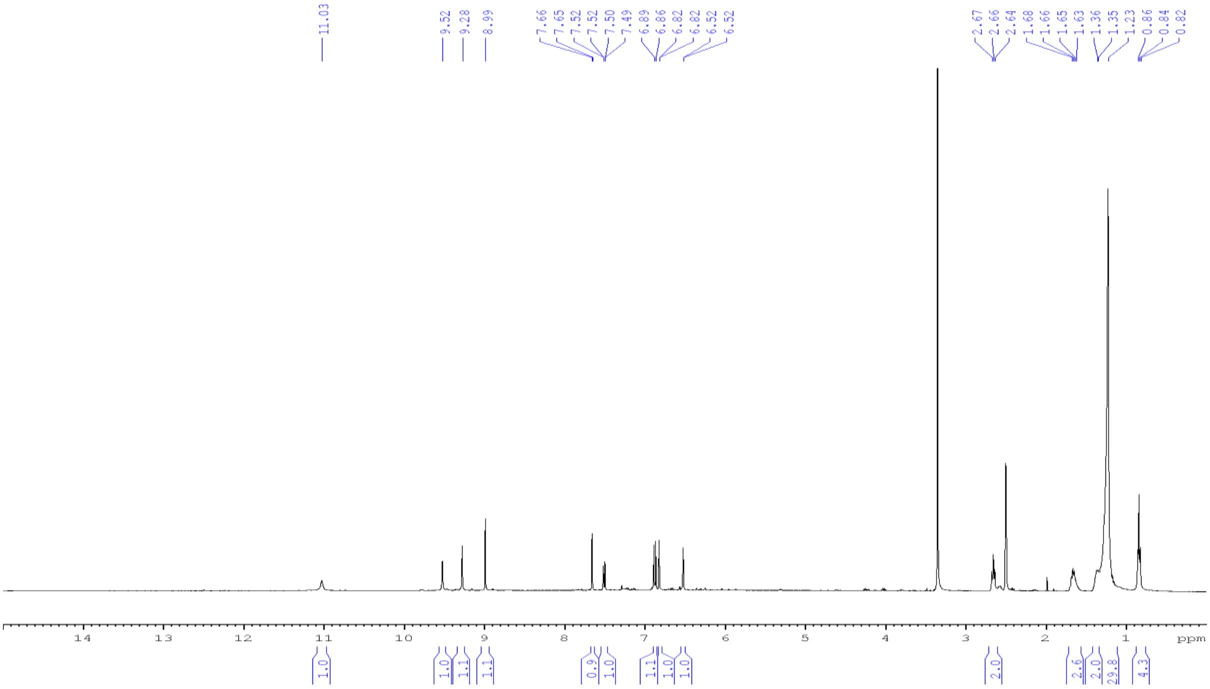

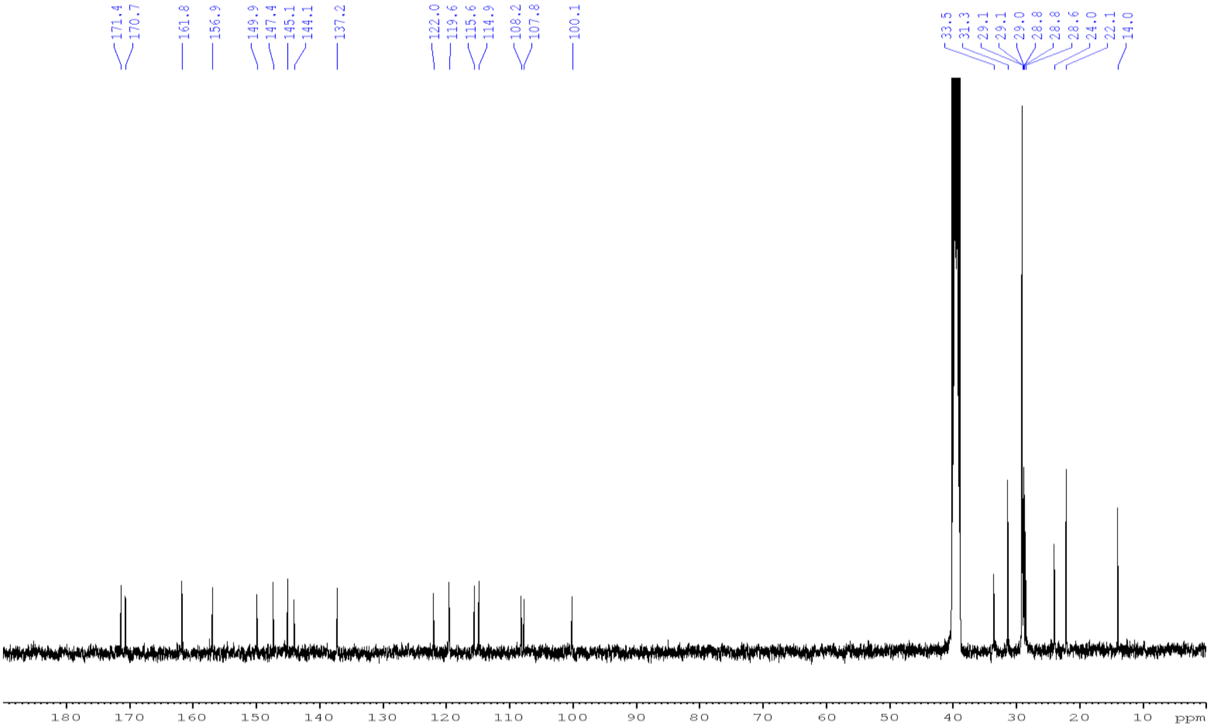

2-(3',4'-Dihydroxyphenyl)-3,7-dihydroxy-4-oxo-4H-chromen-5-yl methyl succinate (5g)

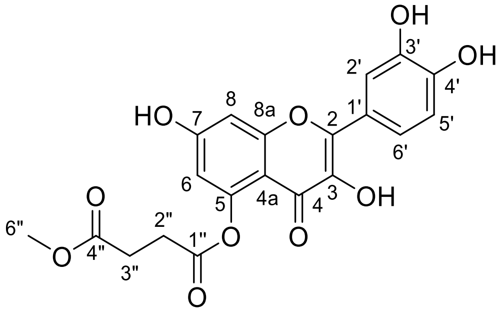

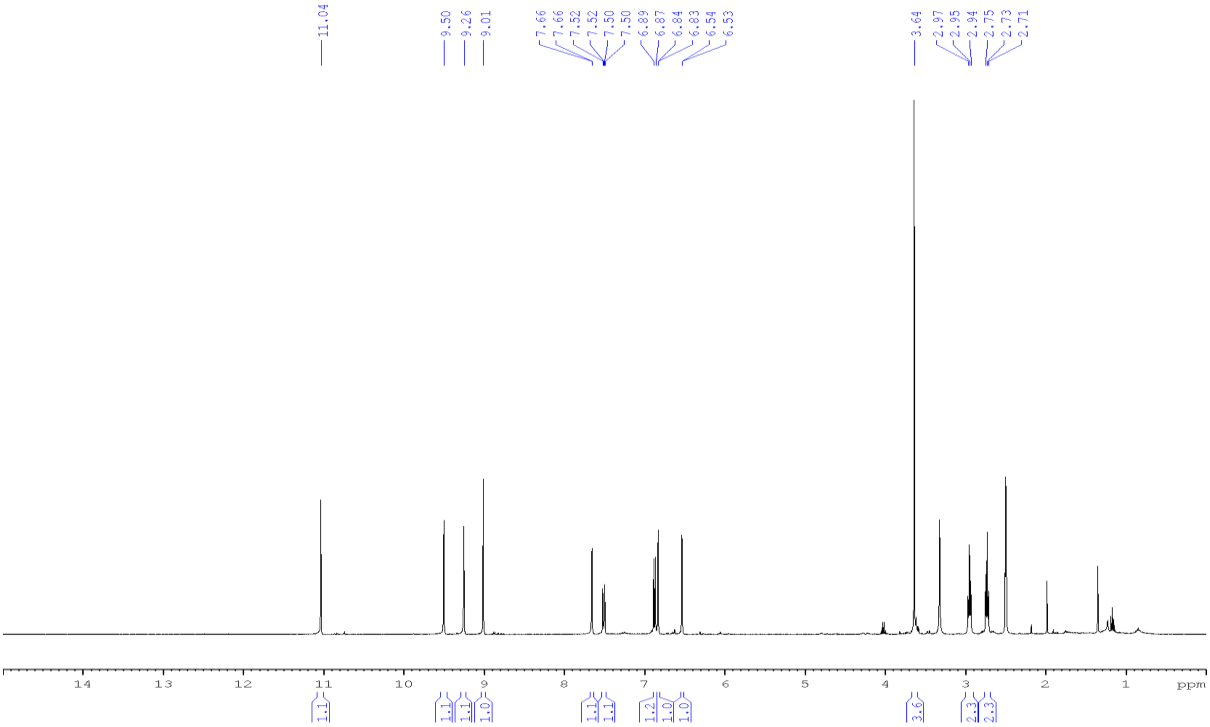

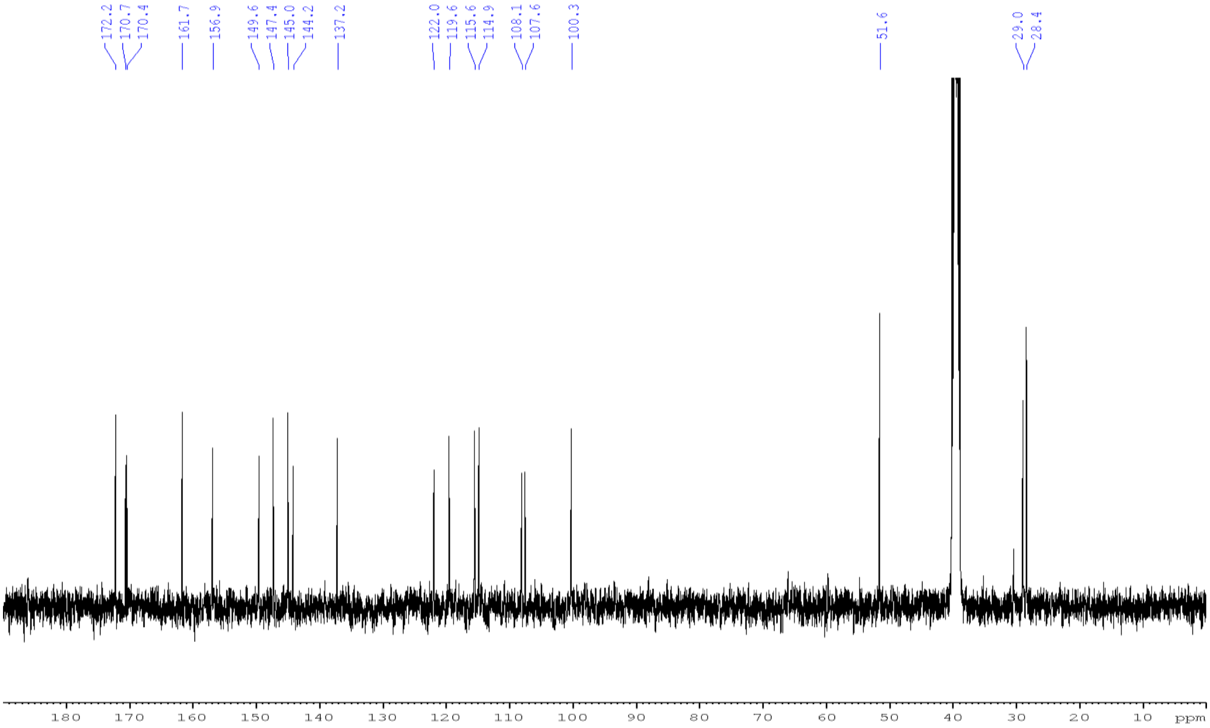

Radical scavenging activity data

Figure S1: Trolox standard curves against ABTS

	Trolox (μM)	% ABTS Scavenged	Standard Curve
**First Assay**	40	1.71%	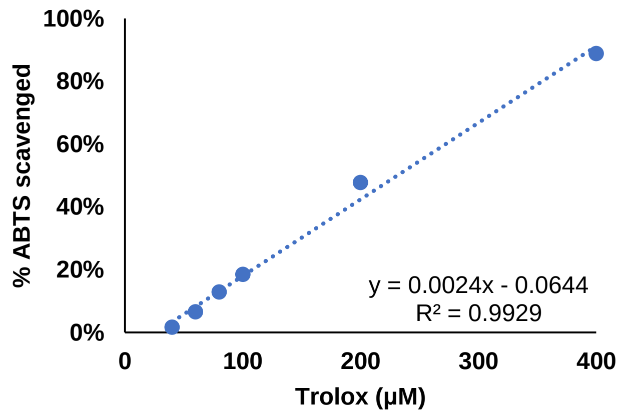
	60	6.56%	
	80	12.86%	
	100	18.46%	
	200	47.71%	
	400	88.85%	
**Second Assay**	40	6.92%	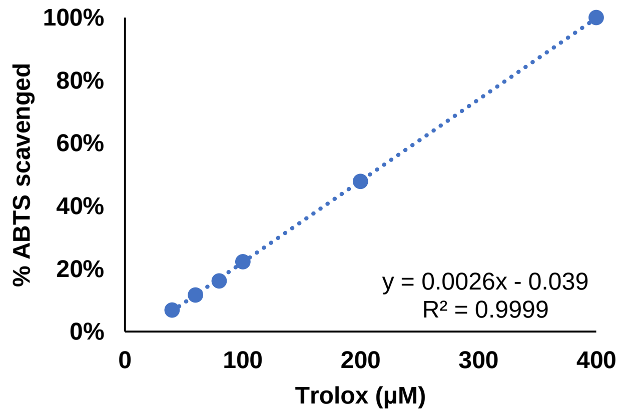
	60	11.69%	
	80	16.17%	
	100	22.30%	
	200	47.90%	
	400	100.00%	
**Third Assay**	40	6.19%	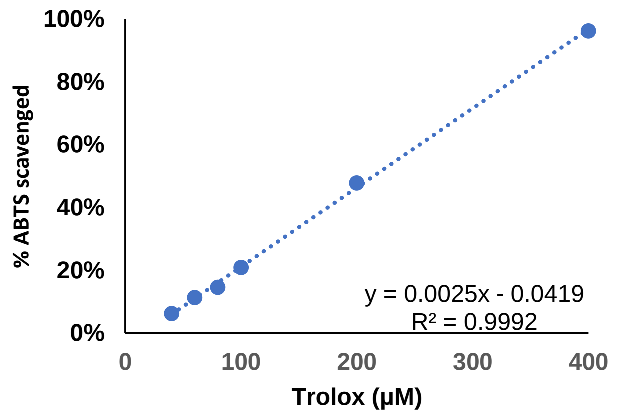
	60	11.25%	
	80	14.55%	
	100	20.85%	
	200	47.78%	
	400	96.19%	

Figure S2: Trolox standard curves against DPPH

	Trolox (µM)	% DPPH Scavenged	Standard Curve
**First Assay**	80	3.48	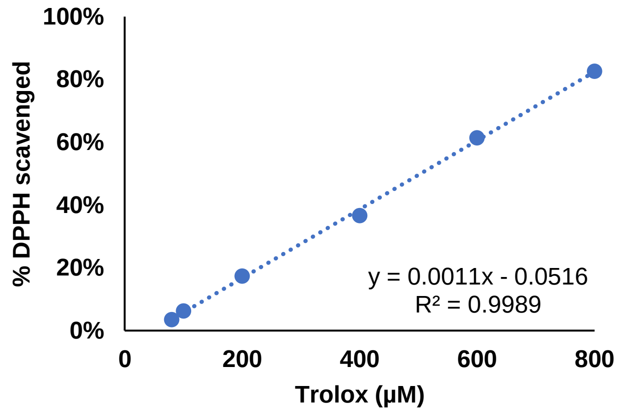
	100	6.26	
	200	17.41	
	400	36.67	
	600	61.45	
	800	82.62	
**Second Assay**	80	3.54	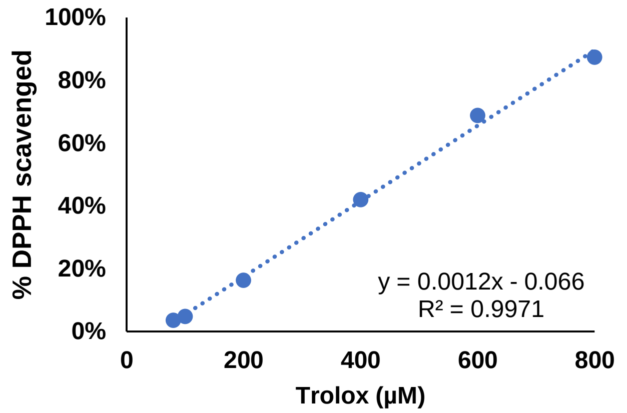
	100	4.75	
	200	16.24	
	400	41.98	
	600	68.74	
	800	87.34	
**Third Assay**	80	4.17	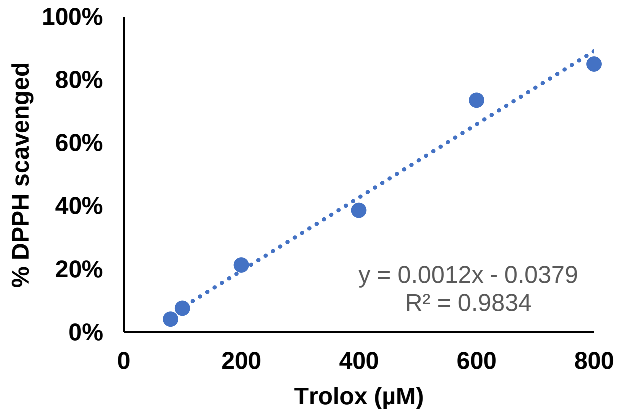
	100	7.65	
	200	21.35	
	400	38.68	
	600	73.57	
	800	85.09	

Figure S3: Radical scavenging activity of quercetin and quercetin derivatives against ABTS

	µM	First Assay	Second Assay	Third Assay	Average	SE	*p*-value
		% ABTS Scavenged	% ABTS Scavenged	% ABTS Scavenged			
Quercetin	20	14.78	15.38	13.52	14.56	0.55	
	40	29.51	30.70	27.73	29.31	0.86	
	60	41.78	42.57	43.01	42.45	0.36	
	80	52.29	67.99	53.92	58.07	4.98	
	100	58.59	94.55	68.07	73.74	10.76	
	150	89.81	98.84	91.02	93.22	2.83	
5a	20	13.29	13.17	10.74	12.40	0.83	0.11
	40	26.89	27.70	25.34	26.64	0.69	0.08
	60	41.84	41.94	42.22	42.00	0.11	0.33
	80	56.51	56.92	53.75	55.73	1.00	0.69
	100	68.20	71.96	66.08	68.75	1.72	0.69
	150	92.80	95.29	91.48	93.19	1.12	0.99
5b	20	13.29	10.95	10.74	11.66	0.82	0.05
	40	26.89	25.31	25.34	25.85	0.52	0.04
	60	41.42	36.61	41.31	39.78	1.58	0.23
	80	56.31	49.63	53.03	52.99	1.93	0.42
	100	68.16	62.82	65.57	65.52	1.54	0.53
	150	93.12	86.07	91.39	90.20	2.12	0.44
5c	20	11.80	9.84	8.78	10.14	0.89	0.02
	40	25.72	22.74	23.17	23.87	0.93	0.01
	60	37.47	35.13	36.92	36.50	0.70	0.00
	80	51.65	45.94	49.39	48.99	1.66	0.20
	100	64.37	56.68	63.95	61.67	2.50	0.38
	150	80.35	82.89	84.98	82.74	1.34	0.05
5d	20	16.08	13.25	12.88	14.07	1.01	0.70
	40	31.84	28.31	29.23	29.79	1.06	0.74
	60	47.05	44.68	48.58	46.77	1.13	0.05
	80	60.48	58.27	62.51	60.42	1.22	0.69
	100	72.60	71.66	73.83	72.70	0.63	0.93
	150	98.54	99.77	98.32	98.88	0.45	0.18
5e	20	15.43	15.58	13.86	14.96	0.55	0.64
	40	28.15	29.02	27.56	28.24	0.43	0.35
	60	41.53	42.24	41.07	41.61	0.34	0.16
	80	56.31	55.88	52.86	55.02	1.09	0.61
	100	66.38	73.28	70.31	69.99	2.00	0.76
	150	94.48	88.12	88.99	90.53	1.99	0.48

	μM	First Assay	Second Assay	Third Assay	Average	SE	*p*-value
		% ABTS Scavenged	% ABTS Scavenged	% ABTS Scavenged			
**5f**	**20**	13.26	12.85	12.88	13.00	0.13	0.10
	**40**	27.56	24.39	26.81	26.25	0.96	0.08
	**60**	40.17	39.80	39.57	39.85	0.18	0.01
	**80**	55.60	54.63	52.51	54.25	0.91	0.53
	**100**	66.05	64.24	64.88	65.06	0.53	0.50
	**150**	92.91	90.85	91.51	91.75	0.61	0.66
**5g**	**20**	10.77	13.11	13.16	12.35	0.79	0.09
	**40**	26.53	29.56	29.32	28.47	0.97	0.55
	**60**	39.51	41.61	43.73	41.62	1.22	0.57
	**80**	49.67	55.51	56.84	54.00	2.20	0.51
	**100**	66.48	70.66	72.15	69.76	1.70	0.75
	**150**	90.68	93.10	94.69	92.82	1.17	0.91

Figure S4: Radical scavenging activity of quercetin and quercetin derivatives against DPPH

	μM	First Assay	Second Assay	Third Assay	Average	SE	*p*-value
		% DPPH Scavenged	% DPPH Scavenged	% DPPH Scavenged			
Quercetin	50	8.81	5.16	7.60	7.19	1.07	
	100	19.04	15.97	18.98	18.00	1.01	
	200	36.39	36.34	39.21	37.31	0.95	
	300	52.05	37.17	51.67	46.96	4.90	
	400	66.13	64.18	69.71	66.68	1.62	
	500	77.57	74.99	79.11	77.22	1.20	
5a	50	6.81	2.67	6.63	5.37	1.35	0.35
	100	15.53	13.06	15.51	14.70	0.82	0.07
	200	31.25	28.02	31.85	30.37	1.19	0.01
	300	44.25	49.84	46.46	46.85	1.63	0.98
	400	57.78	61.48	61.76	60.34	1.28	0.04
	500	68.88	70.00	72.00	70.29	0.91	0.01
5b	50	6.84	6.41	6.04	6.43	0.23	0.55
	100	15.96	17.42	16.20	16.53	0.45	0.29
	200	31.22	35.71	33.31	33.41	1.30	0.08
	300	45.33	51.30	50.28	48.97	1.84	0.73
	400	56.26	63.15	67.18	62.20	3.19	0.30
	500	66.63	75.41	79.01	73.68	3.68	0.44
5c	50	7.54	8.57	5.90	7.34	0.78	0.92
	100	15.77	14.70	17.96	16.14	0.96	0.25
	200	28.84	29.53	28.98	29.12	0.21	0.01
	300	41.51	40.80	42.12	41.48	0.38	0.38
	400	50.83	54.65	54.95	53.48	1.33	0.00
	500	63.02	66.12	59.70	62.95	1.85	0.00
5d	50	9.61	9.16	10.78	9.85	0.48	0.12
	100	19.26	22.02	20.79	20.69	0.80	0.11
	200	36.26	42.19	33.79	37.41	2.49	0.97
	300	51.61	58.60	53.71	54.64	2.07	0.25
	400	63.56	71.85	66.64	67.35	2.42	0.83
	500	74.59	81.34	75.17	77.03	2.16	0.94
5e	50	10.33	9.95	10.48	10.26	0.16	0.10
	100	21.06	20.63	19.85	20.51	0.36	0.12
	200	38.39	41.20	34.87	38.15	1.83	0.71
	300	54.91	45.15	51.55	50.54	2.86	0.57
	400	62.42	67.11	64.39	64.64	1.36	0.39
	500	73.02	75.61	71.39	73.34	1.23	0.09

	μM	First Assay<br>% DPPH Scavenged	Second Assay<br>% DPPH Scavenged	Third Assay<br>% DPPH Scavenged	Average	SE	*p*-value
**5f**	**50**	7.37	8.67	5.41	7.15	0.95	0.980
	**100**	17.72	17.01	13.09	15.94	1.44	0.315
	**200**	35.24	37.27	30.39	34.30	2.04	0.278
	**300**	49.17	49.98	49.00	49.38	0.30	0.670
	**400**	60.94	62.50	64.23	62.56	0.95	0.109
	**500**	73.99	75.80	76.29	75.36	0.70	0.267
**5g**	**50**	7.01	9.07	8.22	8.10	0.60	0.512
	**100**	17.85	19.00	19.64	18.83	0.52	0.520
	**200**	36.75	36.48	35.97	36.40	0.23	0.440
	**300**	52.17	52.57	50.53	51.76	0.62	0.431
	**400**	65.19	65.67	63.93	64.93	0.52	0.396
	**500**	76.51	79.97	73.31	76.60	1.92	0.799
